# Three New Derivatives of Zopfinol from *Pseudorhypophila Mangenotii* gen. et comb. nov.

**DOI:** 10.3390/jof7030181

**Published:** 2021-03-03

**Authors:** Karen Harms, Andrea Milic, Alberto M. Stchigel, Marc Stadler, Frank Surup, Yasmina Marin-Felix

**Affiliations:** 1 Helmholtz Centre for Infection Research GmbH and German Centre for Infection Research (DZIF), Department Microbial Drugs, Partner Site Hannover-Braunschweig, 38124 Braunschweig, Germany; Karen.Harms@helmholtz-hzi.de (K.H.); andrea.milic@student.uni-tuebingen.de (A.M.);Marc.Stadler@helmholtz-hzi.de (M.S.); 2 Institute of Microbiology, Technische Universität Braunschweig, Inhoffenstraße 7, 38124 Braunschweig, Germany; 3 Mycology Unit, Medical School and Pere Virgili Health Research Institute IISPV, Universitat Rovira i Virgili, C/ Sant Llorenç 21, 43201 Reus, Tarragona, Spain; albertomiguel.stchigel@urv.cat

**Keywords:** Antimicrobials, cytotoxicity, Naviculisporaceae, secondary metabolites, Sordariales, sordarins

## Abstract

*Triangularia mangenotti* was analyzed for the production of secondary metabolites, resulting in the isolation of known zopfinol (**1**) and its new derivatives zopfinol B–C (**2–4**), the 10-membered lactones 7-O-acetylmultiplolide A (**5**) and 8-O-acetylmultiplolide A (**6**), together with sordarin (**7**), sordarin B (**8**), and hypoxysordarin (**9**). The absolute configuration of **1** was elucidated by the synthesis of MPTA-esters. Compound **1** showed antimicrobial activity against the Gram-positive bacteria *Bacillus subtilis* and *Staphylococcus aureus* and the fungus *Mucor hiemalis*. While **4** was weakly antibacterial, **3** showed stronger antibiotic activity against the Gram-positive bacteria and weak antifungal activity against *M. hiemalis* and *Rhodotorula glutinis*. We furthermore observed the cytotoxicity of **1**, **3** and **4** against the mammalian cell lines KB3.1 and L929. Moreover, the new genus *Pseudorhypophila* is introduced herein to accommodate *Triangularia mangenotii* together with several species of *Zopfiella*—*Z. marina*, *Z. pilifera*, and *Z. submersa*. These taxa formed a well-supported monophyletic clade in the recently introduced family Navicularisporaceae, located far from the type species of the respective original genera, in a phylogram based on the combined dataset sequences of the internal transcribed spacer region (ITS), the nuclear rDNA large subunit (LSU), and fragments of the ribosomal polymerase II subunit 2 (*rpb2*) and β-tubulin (*tub2*) genes. *Zopfiella submersa* is synonymized with *P. marina* due to the phylogenetic and morphological similarity. The isolation of zopfinols **1**–**4** and sordarins **7**–**9** confirms the potential of this fungal order as producers of bioactive compounds and suggests these compounds as potential chemotaxonomic markers.

## 1. Introduction

The genus *Triangularia* was recently found to be polyphyletic, and its species were scattered along the phylogenetic tree of the order Sordariales [1,2]. Two years ago, Wang et al. [3] delimited the genus to the type species, together with other species previously placed in the genera *Apiosordaria*, *Podospora*, and *Zopfiella*. Recently, *Triangularia karachiensis* was transferred to the new genus *Lundqvistomyces*, since it was not located in the monophyletic clade comprising *Triangularia* [4]. On the other hand, the genus *Zopfiella* could so far not be correctly delimited due to the lack of type material of the type species *Z. tabulata* [4]. One reference strain of this species was placed with other ones producing ascospores with septate upper cell in the family Lasiosphaeriaceae, suggesting that this is the right monophyletic lineage representing the genus [3,4]. Therefore, other species of *Zopfiella* not located in this lineage have been transferred to other genera, e.g., *Z. longicaudata* and *Z. tetraspora* to *Triangularia* [3,4], and *Z. tanzaniensis* to *Lundqvistomyces* [4]. However, a large number of species that are still placed in *Triangularia* and *Zopfiella* need a relocation in different genera to achieve a more natural classification.

Numerous members of the Sordariales are being tested for the production of novel biologically active compounds during the course of an ongoing project, since this group of fungi has been demonstrated to contain prolific producers [5,6]. A prominent example is the production of the antimycotic sordarins by several taxa belonging to this order, e.g., *Rhypophila pleiospora* [7] and *Zopfiella marina* [8]. Moreover, several strains of *Jugulospora* already tested by us demonstrated to be profuse producers of secondary metabolites, as exemplified by the recent report of seven bioactive xanthoquinodin derivatives [9].

Investigations on the secondary metabolism of the type strain of *T. mangenotii* led to the isolation of a plethora of bioactive metabolites, including three different sordarins—sordarin, sordarin B, and hypoxysordarin—zopfinol, and three new derivatives of this. The structures of these three new compounds and the absolute configuration of zopfinol, which was uncertain until now, were elucidated by one-dimensional and two-dimensional nuclear magnetic resonance (1D- and 2D-NMR) spectroscopy. Details of the isolation, structure elucidation, antimicrobial activity, and cytotoxicity of all the isolated compounds are presented herein. Moreover, the new genus *Pseudorhypophila* is introduced to accommodate *T. mangenotii*, which was located far from the monophyletic clade *Triangularia*, together with other three species of *Zopfiella* clustering in the same well-supported clade in the family Naviculisporaceae.

## 2. Materials and Methods

### 2.1. Phylogenetic Study

A phylogenetic analysis based on the combination of sequences of the internal transcribed spacer region (ITS), the nuclear rDNA large subunit (LSU), and fragments of ribosomal polymerase II subunit 2 (*rpb2*) and β-tubulin (*tub2*) genes, and was carried out including sequences of the type strain of *Triangularia mangenotii* and selected members of the Sordariales, with *Camarops amorpha* SMH 1450 as an outgroup (Table 1). Each locus was aligned separately using MAFFT v7 [10] and manually adjusted in MEGA v6.06 [11]. Prior to the concatenation of the four loci, the individual locus phylogenies were checked for conflicts [12,13] and the best evolutionary model for each sequence dataset was calculated using MrModeltest v2.3 [14]. The maximum-likelihood (ML) and Bayesian inference (BI) methods were used in a phylogenetic analysis based on the combined aligned data. The ML analyses employed RAxML on the CIPRES portal (www.phylo.org, accessed on 12 November 2020) using RAxML-HPC BlackBox v8.2.12 with default parameters [15]. The BI was carried out in MrBayes v3.2.1 [16], employing the Markov chain Monte Carlo sampling (MCMC) analysis of four parallel runs of 10 M generations, starting from a random tree topology, and stopping automatically when the average standard deviation of split frequencies fell below 0.01. The sampling frequency was set every 1000 generations and the ‘burn-in’ at 25 %, after which the likelihood values were stationary, and the remaining trees were used to calculate posterior probabilities. Bootstrap support (bs) > 70 and posterior probability values (pp) > 0.95 were considered significant [17].

### 2.2. Fermentation and Extraction

The fungus was grown in yeast malt agar (YM agar; malt extract 10 g/L, yeast extract 4 g/L, d-glucose 4 g/L, agar 20 g/L, pH 6.3 before autoclaving [28]) at 23 °C. Once the fungus was grown, the cultures were cut into small pieces using a cork borer (1 × 1 cm) and five of these pieces were placed into a 200 mL Erlenmeyer flask containing 100 mL of yeast-malt extract broth (YM broth; malt extract 10 g/L, yeast extract 4 g/L, d-glucose 4 g/L, pH 6.3 before autoclaving) under shake conditions at 140 rpm at 23 °C. After 20 days, 10 flasks of 500 mL containing BRFT medium [brown rice 28 g as well as 0.1 L of base liquid (yeast extract 1 g/L, sodium tartrate 0.5 g/L, KH_2_PO_4_ 0.5 g/L [29])] were inoculated with 6 mL of the seed culture, and incubated for 15 days at 23 °C.

For the compound extraction, the solid cultures in BRFT were covered with acetone, and sonicated in an ultrasonic bath for 30 min at 40 °C. Paper filters were used to separate the acetone from the mycelium, and the latter was again subjected to the same sonication and separation procedure. Both acetone extracts were combined and dried in vacuo at 40 °C. The remaining aqueous residue was diluted with the same amount of ethyl acetate (EtOAc) and extracted twice. The crude extract obtained after drying in vacuo at 40 °C was solved in methanol (MeOH) and extracted twice against one-part methanol-water (distilled water, methanol 1:1) and one-part heptane. Finally, the aqueous phase was again diluted with the same amount of EtOAc and extracted twice. The extracts were combined, dried in vacuo at 40 °C and weighed. Crude extract yield was 2230 mg.

### 2.3. Isolation of Compounds **1**–**9**

For isolation of **1**–**9**, the crude extract from BFRT medium in MeOH was portioned to 5 × 450 mg and separated using a PLC 2250 preparative HPLC system (Gilson, Middleton, WI, USA) with a Gemini^®^ 10u C_18_ 110Å column (250 × 21.20 mm, 10 µm; Phenomenex, Torrance, CA, USA) as the stationary phase and in the following conditions: solvent A: H_2_O + 0.1% formic acid, solvent B: ACN + 0.1% formic acid; flow: 45 mL/min, fractionation: 15 mL, gradient: increase from 5% B to 23% B for 10 min, followed by an increase to 27% B in 25 min, then increase to 45% B in 5 min, followed by an increase to 47% in 25 min, then increase to 100% in 7 min, and a final isocratic step of 100% B for 5 min. This yielded the pure fractions of compound **5** (65 mg, *t*_R_ = 34–35 min), compound **6** (613 mg, *t*_R_ = 39–41 min), compound **2** (23 mg, *t*_R_ = 61.5–62.5 min), compound **8** (18.5 mg, *t*_R_ = 66.5–67.5 min), compound **7** (140.5 mg, *t*_R_ = 73.5–74.5 min), as well as the yet impure fractions 7, 9, 10, and 11.

Compound **1** (6.9 mg, *t*_R_ = 23.5–25 min) was obtained from purification of fraction 9, and **9** (1.92 mg, *t*_R_ = 36–37 min) from purification of fraction 11 in the same HPLC system with the same solvents, using XBridge^®^ Prep C_18_ 5 μm OBD^TM^ (250 × 19 mm, 5 µm; Waters, Milford, MA, USA) as the stationary phase with a flow rate of 15 mL/min and a fractionation of 5 mL. The HPLC gradient for the purification of fraction 9 is as follows: increase from 33% B to 43% B for 15 min, followed by an increase to 50% B in 30 min, then increase to 100% B in 10 min, and a final isocratic elution of 100% B for 5 min. The HPLC gradient for the purification of fraction 11 consists of an increase from 45% B to 50% B for 10 min, followed by an increase to 55% B in 30 min, then an increase to 100% B in 7 min, and a final isocratic elution of 100% B for 5 min.

Fraction 7 and 10 were further separated using an Agilent 1200 Infinity Series HPLC-UV system (Agilent Technologies, Santa Clara, CA, USA) with a Nucleodur 100-10 C18ec (250 × 10 mm, 10 µm; Macherey-Nagel, Düren, Germany) as the stationary phase and the following conditions: solvent A: H_2_O + 0.1% formic acid, solvent B: ACN + 0.1% formic acid; flow: 5 mL/min, fractionation: 2.5 mL. Compound **3** (1.75 mg, *t*_R_ = 19.5–20.5 min) was obtained from fraction 10 with the following gradient: an increase from 30% B to 40% B for 7 min, then an increase to 60 % B in 30 min, an increase to 100% B in 7 min, and a final isocratic step of 100% B for 7 min. Compound **4** (0.8 mg, *t*_R_ = 22–23 min) was obtained from fraction 7 with the following gradient: an increase from 25% B to 38% B for 7 min, followed by an increase to 43% B in 20 min, then an increase to 100% B in 7 min, and a final isocratic step of 100% B for 5 min.

### 2.4. Chromatography and Spectral Methods

Electrospray ionization mass spectra (ESI-MS) were recorded on an UltiMate^®^ 3000 Series UHPLC system (Thermo Fisher Scientific, Waltman, MA, USA) connected to an ion trap mass spectrometer (ESI-Ion Trap-MS, amazon speed, Bruker, Billerica, MA, USA), utilizing a C18 Acquity^®^ UPLC BEH column (2.1 × 50 mm, 1.7 m; Waters, Milford, MA, USA), solvent A: H_2_O + 0.1% formic acid, solvent B: ACN + 0.1% formic acid, gradient 5% B for 0.5 min, increasing to 100% B in 19.5 min, maintaining 100% B for a further 5 min, flow rate 0.6 mL/min, UV/Vis detection 190–600 nm.

High-resolution electrospray ionization mass spectra (HR-ESI-MS) were acquired with an Agilent 1200 Infinity Series HPLC-UV system (Agilent Technologies, Santa Clara, CA, USA) connected to a time-of-flight mass spectrometer (ESI-TOF-MS, Maxis, Bruker, Billerica, MA, USA) (scan range 100–2500 m/z, rate 2 Hz, capillary voltage 4500 V, dry temperature 200 °C), using the same HPLC conditions described in ESI-MS measurements.

The 1D and 2D nuclear magnetic resonance (NMR) spectra were recorded with an Avance III 700 spectrometer with a 5 mm TXI cryoprobe (Bruker, 1H NMR: 700 MHz, 13C: 175 MHz, Billerica, MA, USA) and an Avance III 500 (Bruker, 1H NMR: 500 MHz, 13C: 125 MHz, Billerica, MA, USA) spectrometer. The chemical shifts *δ* were referenced to the solvents DMSO-*d*_6_ (^1^H, *δ* = 2.50 ppm; ^13^C, *δ* = 39.51 ppm), and pyridine-*d*_5_ (^1^H, *δ* = 7.22 ppm; ^13^C, *δ* = 123.87 ppm.

Optical rotations were taken with an MCP 150 circular polarimeter at 20 °C (Anton Paar, Graz, Austria) and UV/Vis spectra with a UV-2450 spectrophotometer (Shimadzu, Kyoto, Japan), both in methanol solution MeOH.

### 2.5. Spectral Data

#### 2.5.1. Zopfinol (**1**)

Yellow oil; [α]^20^_D_ + 19° (c 0.001, MeOH); UV (MeOH) λ_max_ (log ε) 296.5 (3.5), 256.5 (4.0), 217.5 (4.3); ^1^H-NMR and ^13^C-NMR see Table 1; ESI-MS: *m/z* 339.16 (M − H)^−^ and 363.17 (M + Na) ^+^; high-resolution electrospray ionization mass spectrometry (HRESIMS) *m/z* 363.1333 (M + Na)^+^ (calculated for C_18_H_25_ClNaO_4_, 363.1339).

#### 2.5.2. Zopfinol B (**2**)

Yellow oil; [α]^20^_D_ + 22° (c 0.001, MeOH); UV (MeOH) λ_max_ (log ε) 296.0 (3.6), 252.5 (4.1), 218.0 (4.5); ^1^H- NMR and ^13^C-NMR see Table 1; ESI-MS: *m/z* 305.07 (M—H)^−^ and 271.08 (M—2H_2_O)^+^; high-resolution electrospray ionization mass spectrometry (HRESIMS) *m/z* 307.1276 (M + H)^+^ (calculated for C_18_H_27_O_4_, 307.1909).

#### 2.5.3. Zopfinol C (**3**)

Colourless-to-white crystals; [α]^20^_D_ + 13° (c 0.001, MeOH); UV (MeOH) λ_max_ (log ε) 297.0 (3.6), 256.0 (4.1), 217.5 (4.4); ^1^H- NMR and ^13^C-NMR see Table 1; ESI-MS: *m/z* 341.18 (M − H)^−^ and 307.15 (M—2H_2_O)^+^; high-resolution electrospray ionization mass spectrometry (HRESIMS) *m/z* 365.1491 (M + Na)^+^ (calculated for C_18_H_27_NaClO_4_, 365.1496).

#### 2.5.4. Zopfinol D (**4**)

Yellow oil; [α]^20^_D_ + 22° (c 0.0005, MeOH); UV (MeOH) λ_max_ (log ε) 295.0 (3.5), 252.5 (3.9), 217.5 (4.3); ^1^H-NMR and ^13^C-NMR see Table 1; ESI-MS: *m/z* 307.15 (M − H)^−^ and 273.11 (M—2H_2_O)^+^; high-resolution electrospray ionization mass spectrometry (HRESIMS) *m/z* 331.1878 (M + Na)^+^ (calculated for C_18_H_28_NaO_4_, 331.1885). 

#### 2.5.5. 7-O-Acetylmultiplolide A (**5**)

Colourless oil; [α]^20^_D_ + 46° (c 0.0005, MeOH); UV (MeOH) λ_max_ (log ε) 202.0 (3.7); ^1^H-NMR and ^13^C-NMR were in good agreement with the literature [30]; ESI-MS: *m/z* 278.99 (M + Na)^+^; high-resolution electrospray ionization mass spectrometry (HRESIMS) *m/z* 279.0837 (M + Na)^+^ (calculated for C_12_H_16_NaO_6_, 279.0845).

#### 2.5.6. 8-O-Acetylmultiplolide A (**6**)

Colourless oil; [α]^20^_D_ + 42° (c 0.001, MeOH); UV (MeOH) λ_max_ (log ε) 202.0 (3.7); ^1^H-NMR and ^13^C-NMR were in good agreement with the literature [30]; ESI-MS: *m/z* 278.98 (M + Na)^+^; high-resolution electrospray ionization mass spectrometry (HRESIMS) *m/z* 279.08418 (M + Na)^+^ (calculated for C_12_H_16_NaO_6_, 279.0845).

#### 2.5.7. Sordarin (**7**)

White powder; [α]^20^_D_ − 35° (c 0.001, MeOH); UV (MeOH) λ_max_ (log ε) 203.0 (3.7); ^1^H-NMR and ^13^C-NMR were in good agreement with the literature [31]; ESI-MS: *m/z* 491.21 (M − H)^−^ and 493.19 (M + H)^+^; high-resolution electrospray ionization mass spectrometry (HRESIMS) *m/z* 493.2787 (M + H)^+^ (calculated for C_27_H_41_O_8_, 493.2801).

#### 2.5.8. Sordarin B (**8**)

White powder; [α]^20^_D_ − 61° (c 0.001, MeOH); UV (MeOH) λ_max_ (log ε) 202.5 (3.7); ^1^H-NMR and ^13^C-NMR were in good agreement with the literature [7]; ESI-MS: *m/z* 491.27 (M − H)^−^ and 493.24 (M + H)^+^; high-resolution electrospray ionization mass spectrometry (HRESIMS) *m/z* 493.2786 (M + H)^+^ (calculated for C_27_H_41_O_8_, 493.2801).

#### 2.5.9. Hypoxysordarin (**9**)

White powder; [α]^20^_D_ + 15° (c 0.001, MeOH); UV (MeOH) λ_max_ (log ε) 210.5 (4.0); ^1^H-NMR and ^13^C-NMR were in good agreement with the literature [32]; ESI-MS: *m/z* 657.35 (M − H)^−^ and 659.33 (M + H)^+^; high-resolution electrospray ionization mass spectrometry (HRESIMS) *m/z* 659.3419 (M + H)^+^ (calculated for C_36_H_51_O_11_, 659.3431).

### 2.6. Derivatization with MTPA

For the preparation of the (*S*)-MTPA ester derivative of **1**, a portion of compound **1** (1.0 mg) was dissolved in pyridine-d_5_ (0.6 mL), transferred into a NMR tube and then (R)-(-)-α-methoxy-α-(trifluoromethyl) phenylacetyl chloride (10 μL) was added. The reaction was monitored by ^1^H NMR followed by the measurement of COSY, TOCSY, HSQC and HMBC NMR spectra. ^1^H NMR (700 MHz, pyridine-*d*_5_): similar to **1**, but δ_H_ 7.43 (8-H), 6.53 (9-H), 6.36 (10-H), 6.14 (11-H), 6.02 (13-H), 5.55 (12-H), 5.44 (1-H), 1.96 (14-H_2_), 1.25 (15-H_2_), 1.20 (17-H_2_), 1.16 (16-H_2_), 0.82 (18-H_3_). 

The (*R*)-MTPA ester was prepared in the same manner by the addition of 10 µL of (*S*)-MTPA chloride: ^1^H NMR (700 MHz, pyridine-*d*_5_): similar to **1**, but δ_H_ 7.32 (8-H), 6.26 (9-H), 6.26 (10-H), 6.21 (11-H), 6.17 (13-H), 5.81 (12-H), 5.44 (1-H), 2.02 (14-H_2_), 1.29 (15-H_2_), 1.18 (17-H_2_), 1.15 (16-H_2_), 0.79 (18-H_3_).

### 2.7. Biological Testing

Isolated compounds were tested for their antimicrobial activity against five fungi (*Candida albicans*, *Mucor hiemalis*, *Rhodotorula glutinis*, *Schizosaccharomyces pombe* and *Wickerhamomyces anomalus*), four Gram-positive bacteria (*Bacillus subtilis*, *Micrococcus luteus*, *Mycobacterium smegmatis* and *Staphylococcus aureus*) and three Gram-negative bacteria (*Chromobacterium violaceum*, *Escherichia coli* and *Pseudomonas aeruginosa*), using nystatin as a positive control against all the tested fungi and oxytetracycline against all the bacteria, except for *My. smegmatis* and *Ps. aeruginosa*, against which kanamycin and gentamycin were used, respectively. Moreover, the cytotoxicity of the compounds against two different mammalian cell lines—human endocervical adenocarcinoma KB 3.1 and mouse fibroblasts L929—were determined by the MTT method using epothilone B as the positive control. Both biological assays were performed following the protocols described by Becker et al. [33].

## 3. Results

### 3.1. Phylogenetic Analysis

**Figure 1:**
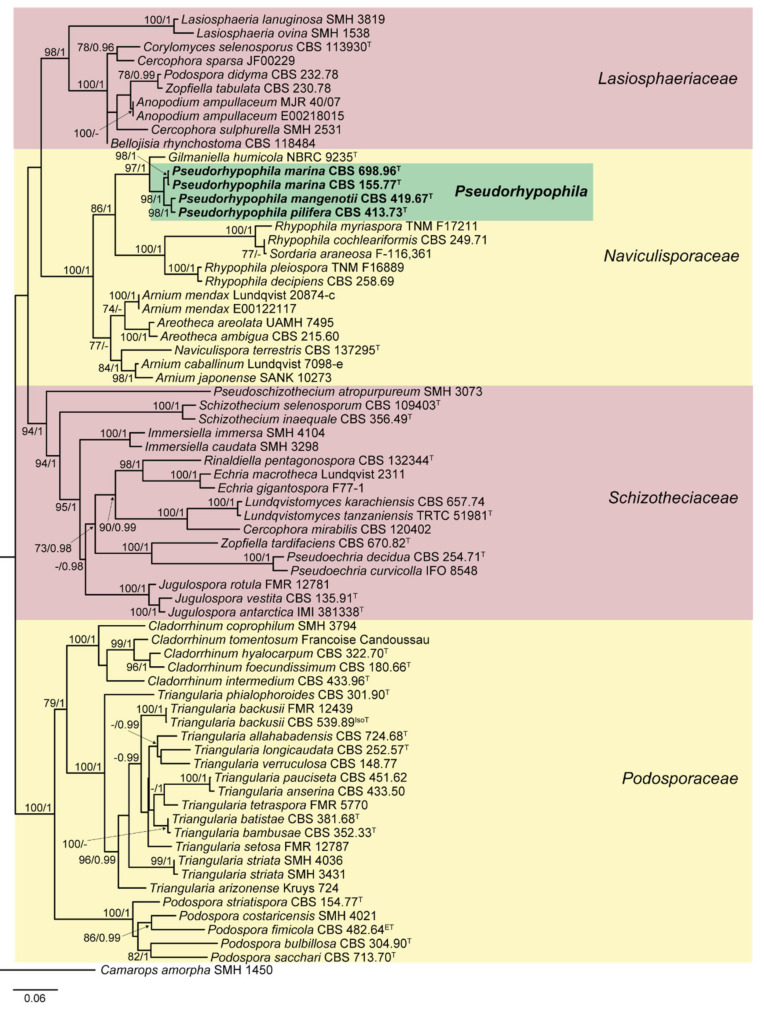
Randomized axelerated maximum likelihood (RAxML) phylogram obtained from the combined sequences of the internal transcribed spacer region (ITS), the nuclear rDNA large subunit (LSU), and fragments of ribosomal polymerase II subunit 2 (*rpb2*) and β-tubulin (*tub2*) genes of selected strains belonging to the families Lasiosphaeriaceae, Naviculisporaceae, Podosporaceae, and Schizotheciaceae, using *Camarops amorpha* SMH 1450 as outgroup. Bootstrap support values ≥ 70 / Bayesian posterior probability scores ≥ 0.95 are indicated along branches. Branch lengths are proportional to distance. Novel taxa proposed in the present study are in **bold**. Ex-epitype, ex-isotype, and ex-type strains of the different species are indicated with ^ET^, ^IsoT^, and ^T^, respectively. Different background colors have been used to highlight the major clades.

The lengths of the individual alignments used in the combined dataset were 634 bp (ITS), 891 bp (LSU), 972 bp (*rpb2*) and 618 bp (*tub2*), and the final total alignment was 3115 bp. The phylogentic tree obtained from the RAxML analysis of the combined dataset, including bootstrap support and Bayesian posterior probability at the nodes, is shown in Figure 1. The RAxML tree obtained agreed with the topology of the tree generated by the Bayesian analysis. The ex-type strain of *Triangularia mangenotii* was located in the Naviculisporaceae clade, forming a well-supported clade (100% bs/1 pp) independent from the other lineages of the family, together with the type strains of *Zopfiella marina*, *Z. pilifera* and *Z. submersa*. However, the monophyletic lineage representing the genus *Triangularia* was placed in the Podosporaceae clade, while the type species of *Zopfiella*, *Z. tabulata* was located in the Lasiosphaeriaceae clade. Therefore, the new genus *Pseudorhypophila* is introduced herein to accommodate these four taxa. Additionally, the close phylogenetic distance between *Z. marina* and *Z. submersa* suggested that these could indeed represent the same taxa. The nucleotide similarity of the *rpb2* sequences of both taxa was 99.88%, while that of the ITS sequences was 99.78% (the only difference was due to the presence of an indeterminable base-pair in one of the sequences). The same occurred in the LSU sequence comparison, in which the similarity was only 97.43% but the differences were due to indeterminate nucleotide positions in the sequences of *Z. marina*. The nucleotide similarity of *tub2* sequences (a fragment different from the one used in the present phylogenetic study; GenBank acc. numbers MK926951 and MK926953) was also 100%. Therefore, and in accordance with phenotype-derived data, the synonymy of both species is proposed.

### 3.2. Taxonomy

***Pseudorhypophila*** Y. Marín and Stchigel, **gen. nov.** MycoBank MB838466.

Type species: *Pseudorhypophila mangenotii* (Arx & Hennebert) Y. Marín & Stchigel.

Etymology: Based on the phylogenetic relation to *Rhypophila*.

Ascomata non-ostiolate or ostiolate, superficial or immersed, black, globose to subglobose, or ovate to pyriform, almost glabrous or covered by short or long, flexuous hairs; neck short, cylindrical to conical, covered with small black papillae. Asci clavate to cylindrical, stipitate, 4–8-spored, with a small apical ring sometimes indistinct. Periphyses present or absent. Paraphyses present or absent, septate, hyaline. Ascospores biseriate, two-celled; upper cell narrowly conical, acuminate towards apex and rounded at base, or ovoid to limoniform with somewhat truncate base, olivaceous brown to dark brown, with an apical or subapical germ pore, sometimes with a distinct apical appendage; lower cell remaining hyaline, or sometimes becoming pale olivaceous brown or pale brown, occasionally dark brown, cylindrical and straight or curved, or hemisphaerical, or at first broadly obconical and then becoming flattened at apex; gelatinous sheats sometimes present, hyaline, thin. Conidia holoblastic, sessile, borne singly along the vegetative hyphae, hyaline, spherical to subspherical, or ovate to elongate, smooth-walled.

Notes: *Pseudorhypophila* is related to *Gilmaniella* and *Rhypophila*. The former genus produces the humicola-like asexual morph characterized by the production of dark brown, spherical conidia with marked apical germ pores and borne singly or in clusters of up to four [34], while the new genus *Pseudorhypophila* produces a chrysosporium-like asexual morph, and the asexual morph is absent in *Rhypophila* [4]. *Rhypophila* differs from *Pseudorhypophila* by the production of ascomata with elongate, tuberculate projections in the neck, while these are mostly non-ostiolate ascomata in the new genus. Moreover, *Rhypophila* is characterized by having mostly more than eight-spored asci and ascospores with lower cell as long as, or longer, than the upper cell.

*Zopfiella submersa* was introduced by Guarro et al. [35] in 1997. These authors discussed the similarity of this taxon with *Zopfiella marina*, which was introduced before by Furuya and Udagawa [36]. The main differences between both species according to reference [36] were the presence of a sexual morph and ascospore with an apical pore in the upper cell in *Z. marina*, whereas the asexual morph is absent and the upper cell of the ascospores have a subapical pore in *Z. submersa*. Both taxa were isolated only from aquatic environments in Asia (China and Iraq). Whereas *Z. marina* was found in marine mud (in depth of 120 m), *Z. submersa* was reported from dead culms of *Arundo donax* submerged in a river. Due to the scarce molecular and morphological differences between both taxa, we proposed here their synonymy under the new combination *P. marina*. The other two species of the genus—*P. mangenotii* and *P. pilifera*—are also closely related to each other, but these showed only a 98.04 % nucleotide similarity of the *rpb2* sequences. Both species are characterized by ascospores with conical upper cells [37], but these can be easily distinguished by the ascomata, being ostiolate in *P. mangenotii* [38] and non-ostiolate in *P. pilifera*, and by the presence of an asexual morph in the latter [39].

**Key to species of *Pseudorhypophila*.**

1. Ostiolate ascomata................................................................................*P. mangenotii*

1. Non-ostiolate ascomata............................................................................................2

2. Ascospores with upper and lower cell conical........................................*P. pilifera*

2. Ascospores with upper cell ovoid to limoniform, and lower cell cylindrical....

..........................................................................................................................*P. marina*

***Pseudorhypophila mangenotii*** (Arx and Hennebert) Y. Marín and Stchigel, **comb. nov.** MycoBank MB838467.

Basionym: *Triangularia mangenotii* Arx and Hennebert, Bull. Trimestriel Soc. Mycol. France 84: 423. 1969.

***Pseudorhypophila marina*** (Furuya and Udagawa) Y. Marín and Stchigel, **comb. nov.** MycoBank MB838468.

Basionym: *Zopfiella marina* Furuya and Udagawa, J. Jap. Bot. 50: 249. 1975. 

Synonym: *Zopfiella submersa* Guarro, Al-Saadoon, Gené and Abdullah, Mycologia 89: 958. 1997.

***Pseudorhypophila pilifera*** (Udagawa and Furuya) Y. Marín and Stchigel, **comb. nov.** MycoBank MB838469.

Basionym: *Zopfiella pilifera* Udagawa and Furuya, Trans. Mycol. Soc. Japan 13: 255. 1972.

### 3.3. Structure Elucidation of Compounds **1**–**4**

Zopfinol (**1**) [40], three novel derivatives of zopfinol (**2**–**4**), 7-O-acetylmultiplolide A (**5**) [30], 8-O-acetylmultiplolide A (**6**) [30,41], sordarin (**7**) [42], sordarin B (**8**) [7], and hypoxysordarin (**9**) [32] were isolated from the 2230 mg of crude extract obtained from the fermentation in rice of the ex-type strain of *Pseudorhypophila mangenotii* (Figure 2 and Figure 3) by preparative HPLC. Their structures were elucidated by 1D- and 2D-NMR spectroscopy (Supplementary Figures S1–S26).

**Figure 4:**
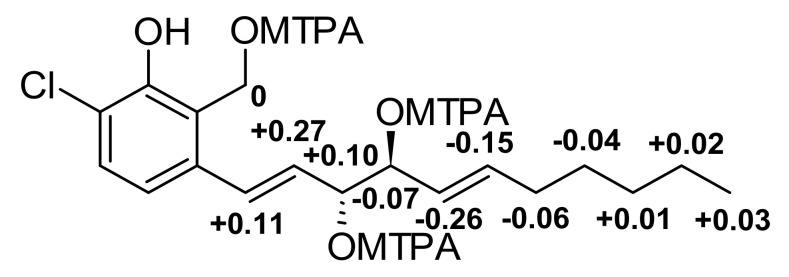
Δδ^SR^ values for MPTA esters of **1** diagnostic for 10*R*,11*S*.

Compound **1** was obtained as a yellow oil and its molecular formula was established as C_18_H_25_ClO_4_ (six degrees of unsaturation) according to the quasimolecular ion peak cluster at *m/z* 363.1333 (M + Na)^+^ in the HRESIMS spectrum. ^1^H and HSQC spectra (Table 2) of **1** revealed the presence of one methyl, two oxymethines, six olefinic/aromatic methines as well as five methylenes, one of which being an oxymethylene. The carbon spectrum revealed the further presence of four aromatic carbon atom-devoid bound protons. Using COSY and TOCSY data, the long side chain CH–8 to C–18 was assembled. A literature search within the dictionary of natural products with this information identified **1** as the known compound zopfinol [40]. Since no stereochemistry has been assigned for **1** to date, we addressed this issue. However, no ^2^*J*_C10H11_ and ^2^*J*_C11H10_ coupling constants were observed in the HSQC-Hecade and *J*-HMBC experiments, so the J-based configurational method gave equivocal results. Nevertheless, the synthesis of multi-MTPA esters of **1** yielded a diagnostic Δδ^SR^ sign distribution pattern. The positive values for 8–H/9–H/10–H in addition to the negative ones for 11–H/12–H/13–H/14–H_2_/15–H_2_ is characteristic for 1,2-diols with *R,S* absolute stereochemistry [43] (Figure 4). Consequently, we assigned a 10*R*,11*S* absolute configuration for **1**.

Compound **2** was obtained as a yellow oil. The molecular ion cluster at *m/z* 307.1276 [M + H]^+^ in the HRESIMS spectrum indicated that the molecular formula of **2** was C_18_H_26_O_4_, indicating the substitution of the chlorine by a hydrogen atom. This observation was confirmed by the additional aromatic olefin signal for 4–H in the ^1^H and HSQC spectra of **2**. Since other NMR data including coupling constants are virtually identical to **1**, a common 10*R*,11*S* was assigned for **2**, too. Consequently, **2** was elucidated as dechlorozopfinol and named zopfinol B.

Compound **3** was obtained as colorless-to-white crystals. The molecular ion cluster at *m/z* 365.1491 [M + Na]+ in the HRESIMS spectrum indicated that the molecular formula is C_18_H_27_ClO_4_. The NMR data of **3** were highly similar to those of **2**, with the key difference being the exchange of the olefinic methines 12–H/13–H by two methylenes. Therefore, we assigned **3** as 12,13-dihydrozopfinol, the name given to it being zopfinol C.

Compound **4** was obtained as a yellow oil and its molecular formula was established as C_18_H_28_O_4_ according to the mass ion peak at *m/z* 331.1878 [M + Na]^+^ in the HRESIMS spectrum, indicating the formal addition of two hydrogens. The key difference in the NMR spectra of **4** compared to **1** was the exchange of the olefinic methines 12–H/13–H by two methylenes. Therefore, we elucidated **4** as dechloro-12,13-dihydrozopfinol, and named it zopfinol D.

### 3.4. Antimicrobial and Cytotoxic Activities of Compounds **1**–**9**

From the nine isolated compounds, only **1**, **3**, **4**, **7** and **9** showed antimicrobial activity (Table 3).

Zopfinol (**1**) and two of its derivatives (**3** and **4**) were active against the Gram-positive bacteria *Bacillus subtilis* and *Staphylococcus aureus*, and compound **3** was also active against *Rhodotorula glutinis*. Compound **1** and **3** showed weak antifungal activity against *Mucor hiemalis*.

On the other hand, compound **7** and **9** showed antifungal activity against *Candida albicans*, even though the activity of **9** was weak. Compound **9** showed a much stronger antifungal activity against *Mucor hiemalis*.

Compound **1**, **3** and **4** showed weak cytotoxic activity against the two different mammalian cell lines tested (Table 4).

## 4. Discussion

Lasiosphaeriaceous genera have been considered polyphyletic since their taxa were scattered in different clades along the Sordariales [1,3,18,22,26]. This was a consequence of the traditional delimitation of the genera based on the ascospore morphology, which resulted in an extremely homoplastic character not useful in predicting the phylogenetic relationships [1,22]. Recent phylogenetic studies based on the ITS, LSU, *rpb2* and *tub2* sequences were focused on the right delimitation of both the polyphyletic family and genera, resulting in the introduction of the monophyletic families Podosporaceae [3], Diplogelasinosporaceae, Naviculisporaceae and Schizotheciaceae [4]. Moreover, some of the genera were properly delimited, such as *Podospora* and *Triangularia* [3]. However, large genera such as *Cercophora* and *Zopfiella* still remain polyphyletic, and other species of the already delimited genera are awaiting a correct taxonomic placement. In that context, the type strain of *T. mangenotii*, which was located in the family Naviculisporaceae and far from the monophyletic clade of *Triangularia* in the Podosporaceae, is currently relocated in the new genus *Pseudorhypophila*, together with other species of *Zopfiella* far from the type species of the genus, *Z. tabulata*, which is located in the Lasiosphaeriaceae. This new genus is characterized by mostly non-ostiolate ascomata and a chrysosporium-like asexual morph. On the other hand, the most phylogenetically related genus, *Gilmaniella*, is characterized by the production of a solely humicola-like asexual morph [34].

Zopfinol (**1**) is a chloratinated phenol with an aliphatic side chain and was isolated before from the marine fungus *Zopfiella marina* [40], which we transferred in the present study to the new genus *Pseudorhypophila*. In addition, the strain we studied produced three new derivatives of zopfinol (**2**–**4**). Compound **1** showed weak antimicrobial activity against *Mucor hiemalis* and *Staphylococcus aureus*, and moderate antibacterial activity against *Bacillus subtilis*. On the other hand, the new derivative **4** showed only a weak activity against the Gram-positive bacteria, *B. subtilis* and *S. aureus*. Compound **3** was moderately active against the same two Gram-positive bacteria, and exhibited weak antifungal activity against *M. hiemalis* and *Rhodotorula glutinis*.

7-*O*-acetylmultiplolide A (**5**) and 8-*O*-acetylmultiplolide A (**6**) are 10-membered lactones, first reported from a *Diaporthe* sp. [30,41], which pertains to the class Sordariomycetes. Both compounds were devoid of antimicrobial activity against the microorganisms tested in the present study. However, compound **6** had shown antifungal activity against *Aspergillus niger*, *Bipolaris maydis*, *Botrytis cinerea*, *Fusarium moniliforme*, *Ophiostoma minus* and *Talaromyces islandicus*, as previously reported by Wu et al. [30]. Surprisingly, compound **5** only showed weak activity against *A. niger*, even though both compounds **5** and **6** differ only in the position of the acetoxy group [30]. Compound **6** was reported to have significant inhibitory activity towards acetylcholinesterase [41], and antihyperlipidemic activity equivalent to that observed in lovastatin, which was used as a positive control [44]. Other ten-membered lactones have been found in *Diaporthe* [30,41,44], as well as in other Sordariomycetes, i.e., *Xylaria multiplex* [45] and *Gilmaniella humicola* [46], the latter of which is also now located in the Naviculisporaceae like *P. mangenotii*. Some of these 10-membered lactones also showed antifungal activity, i.e., multiplolides A and B were active against *Candida albicans* [45]. Moreover, humilactone from *Gilmaniella humicola* showed strong cytotoxic activity [46], which was not observed in the other related compounds mentioned.

The last group of compounds isolated from *P. mangenotii* were the sordarins (**7–9**). Those are a class of natural antifungal agents that act at the protein synthesis level, inhibiting it through their interaction with the elongation factor 2 in eukaryotes (eEF2) [47,48]. This essential enzyme catalyzes the translocation of transfer RNA and messenger RNA after peptide bond formation in the translation process, leading to an inhibition of this step and promoting cell death [49,50]. What makes these compounds have a solely antimycotic activity is the high affinity for fungal eEF2 when it is compared against that of plants or mammals [50]. These compounds are mainly produced by Xylariales, but also by members of Eurotiales, Microascales and Sordariales [8]. In this last order, the taxa reported to produce these kinds of compounds are *Podospora araneosa* [42], *Rhypophila pleiospora* [7] and *Z. marina* [51], which is here transferred to the genus *Pseudorhypophila* (as *P. marina*), all of which are members of the family Naviculisporaceae. Therefore, the production of sordarin and related compounds could be restricted to this family. *Podospora araneosa* clustered in the monophyletic clade of *Rhypophila*, suggesting that it could belong to this genus. However, further studies including the type material of this species need to be carried out to corroborate this hypothesis. Compound **7** was found in cultures of *Podospora araneosa* [42] and **8** in *Rhypophila pleiospora* [7], while **9** was only reported before from *Hypoxylon croceum* [32], which is located in the Xylariales. *Podospora araneosa* also produced hydroxysordarin and neosordarin, which is closely related to **9**, with only small differences in the aliphatic side chain acylating the hydroxyl in the 3′-position of the sordarose moiety [51]. *Pseudorhypophila marina* produces the sordarin derivative known as zofimarin [51], which was demonstrated to have antifungal activity against *Candida albicans*, *C. pseudotropicalis* and *Crytococcus neoformans* [52]. In our antimicrobial study, **8** was not active against any of the microorganisms tested. However, Weber et al. [7] observed antifungal activity against *Nematospora coryli*, *Sporobolomyces roseus* and *Thelebolus nanus*. In the present study, **7** was only active against *C. albicans*, while **9** showed weak activity against *C. albicans* but moderate activity against *M. hiemalis*. The higher antifungal activity of **9** with respect to the other sordarin or sordarin-related compounds was already observed by Davoli et al. [53]. In that work, **9** showed antifungal activity against *Paecilomyces variotii*, *Penicillium notatum*, *Nematospora coryli* and *M. miehei*, while **7** only had activity against the last two fungi. The comparison between the activities of different sordarin derivatives demonstrated that the nature of the side chain plays an important role in the antifungal activity, increasing when there is a 3′-*O*-acyl group and decreasing in the presence of a hydroxymethyl group in the sugar moiety [52,53].

*Pseudorhypophila marina* also produced salicylaldehyde and dihydroisobenzofuran derivatives [54], apart from zopfinols [40] and zopfimarin [51], mentioned before. The structures of these compounds are related to zopfinol, but most of them were not active, except for one of the salicylaldehyde derivatives, which showed weak activity against *Mycobacterium tuberculosis* and *Bacillus cereus* [54]. Other compounds with structures related to zopfinol and its derivatives are the salicylaldehyde sordarial produced by *Neurospora crassa*, which also belongs to the order Sordariales [55], and the pyriculols, which are phytotoxic polyketides produced by the sordariomycete phytopathogenic fungus *Pyricularia oryzae* [56]. Since the phytotoxic pyriculol [57] differs from **2** only in the length of its aliphatic side chain, it would be highly interesting to test the phytotoxicity of zopfinols.

The production of secondary metabolites by the new genus *Pseudorhypophila* could be useful as chemotaxonomic markers, since the zopfinol is produced by different species of *Pseudorhypophila*, but it was not reported in any other taxon. Sordarins seem to be present in different taxa belonging to the family Naviculisporaceae, also being a potential chemotaxonomic marker for this family. Chemotaxonomy could help us in the achievement of a more natural classification of the sordariaceous fungi.

Our work, together with those focused on the screening for bioactive metabolites produced by members of the Sordariales [5,6,9], confirms the potential of this fungal order as a producer of bioactive compounds. In particular, the new genus *Pseudorhypophila* includes species able to produce a plethora of bioactive compounds, including the widely studied antifungal sordarins.

## Figures and Tables

**Figure 2:**
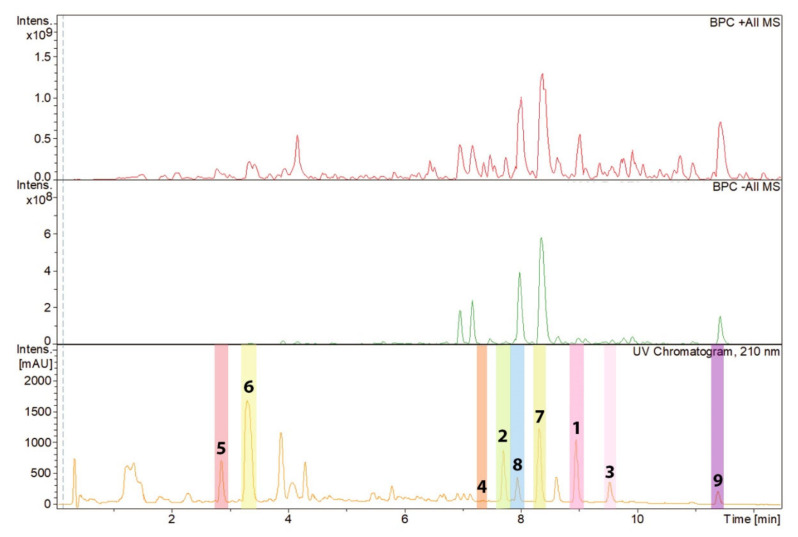
High-performance liquid chromatography (HPLC) chromatogram (210 nm) of the ethyl acetate (EtOAc) extract from *Pseudorhypophila mangenotii* with peaks of the compounds isolated referring to the molecules depicted in Figure 3. The peaks representing compounds **1**–**9** have been highlighted with different colors.

**Figure 3:**
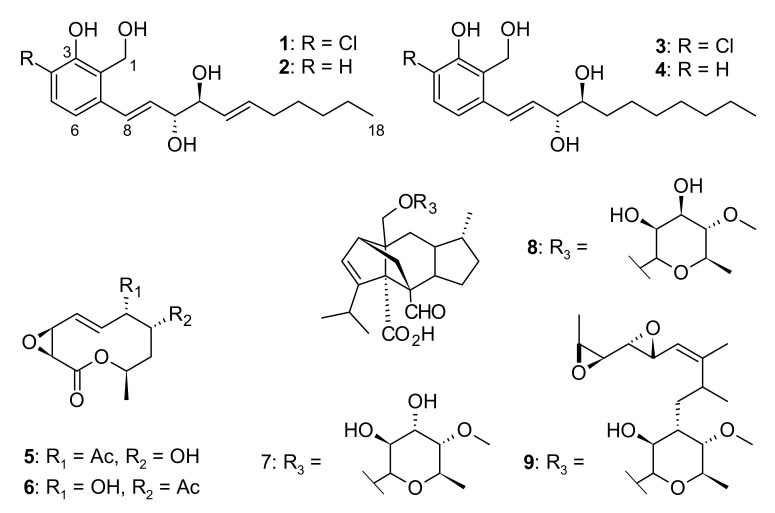
Chemical structures of compounds **1**–**9** isolated from *Pseudorhypophila mangenotii* CBS 419.67.

**Table 1:** Strains of the order Sordariales included in the phylogenetic study. Taxonomic novelties are indicated in bold italic.

Taxa	Strain	GenBank Accession Numbers				Source
		LSU	ITS	*rpb2*	*tub2*	
Anopodium ampullaceum*	MJR 40/07	KF557662	-	-	KF557701	[2]
	E00218015	KF557663	-	-	KF557702	[2]
*Areotheca ambigua*	CBS 215.60	AY999114	AY999137	-	-	[18]
*Areotheca areolata*	UAMH 7495	AY587936	AY587911	AY600275	AY600252	[19]
*Arnium caballinum*	Lundqvist 7098-e	KF557672	-	-	-	[2]
Arnium japonense*	SANK 10273	KF557680	-	KF557713	-	[2]
Arnium mendax*	Lundqvist 20874-c	KF557687	-	KF557716	-	[2]
	E00122117	KF557688	-	KF557717	-	[2]
Bellojisia rhynchostoma*	CBS 118484	EU999217	-	-	-	[20]
*Camarops amorpha*	SMH 1450	AY780054	-	AY780156	AY780093	[1]
*Cercophora mirabilis*	CBS 120402	KP981429	MT784128	KP981611	KP981556	[4]
Cercophora sparsa*	JF 00229	AY587937	AY587912	-	AY600253	[19]
Cercophora sulphurella*	SMH 2531	AY587938	AY587913	AY600276	AY600254	[19]
*Cladorrhinum coprophilum*	SMH 3794	AY780058	-	AY780162	AY780102	[1]
*Cladorrhinum foecundissimum*	CBS 180.66^T^	MK926856	MK926856	MK876818	-	[3]
*Cladorrhinum hyalocarpum*	CBS 322.70^T^	MK926857	MK926857	MK876819	-	[3]
*Cladorrhinum intermedium*	CBS 433.96^T^	MK926859	MK926859	MK876821	-	[3]
*Cladorrhinum tomentosum*	Francoise Candoussau	KF557691	-	-	KF557720	[1]
Corylomyces selenosporus*	CBS 113930^T^	DQ327607	MT784130	KP981612	KP981557	[4,21]
*Echria gigantospora*	F77-1	KF557674	-	-	KF557710	[2]
*Echria macrotheca*	Lundqvist 2311	KF557684	-	-	KF557715	[2]
*Gilmaniella humicola*	NBRC 9235^T^	ITS and LSU sequences only available in https://www.nite.go.jp/nbrc/catalogue/NBRCDispSearchServlet?lang=en (Accessed on 8 November 2020)				
*Immersiella caudata*	SMH 3298	AY436407	-	AY780161	AY780101	[1,22]
*Immersiella immersa*	SMH 4104	AY436409	-	AY780181	AY780123	[1,22]
*Jugulospora antarctica*	IMI 381338^T^	KP981433	-	KP981616	KP981561	[4]
*Jugulospora rotula*	FMR 12781	KP981438	MT784134	KP981621	KP981566	[4]
*Jugulospora vestita*	CBS 135.91^T^	MT785872	MT784135	MT783824	MT783825	[4]
*Lasiosphaeria lanuginosa*	SMH 3819	AY436412	AY587921	AY600262	AY600283	[19,22]
*Lasiosphaeria ovina*	SMH 1538	AF064643	AY587926	AY600287	AF466046	[19,23,24]
*Lundqvistomyces karachiensis*	CBS 657.74	KP981447	MK926850	KP981630	KP981478	[3,4]
*Lundqvistomyces tanzaniensis*	TRTC 51981^T^	AY780081	MH862260	AY780197	AY780143	[1,25]
*Naviculispora terrestris*	CBS 137295^T^	KP981439	MT784136	KP981622	KP981567	[4]
*Podospora bulbillosa*	CBS 304.90^T^	MK926861	MK926861	MK876823	-	[3]
Podospora didyma*	CBS 232.78	AY999100	AY999127	-	-	[18]
*Podospora fimicola*	CBS 482.64^ET^	KP981440	MK926862	KP981623	KP981568	[3,4]
*Podospora sacchari*	CBS 713.70^T^	KP981425	MH859915	KP981607	KP981552	[4,25]
*Podospora striatispora*	CBS 154.77^T^	KP981426	MT784137	KP981608	KP981553	[4]
*Pseudoechria curvicolla*	NBRC 8548	AY999099	AY999122	-	-	[18]
*Pseudoechria decidua*	CBS 254.71^T^	MK926842	MK926842	MK876804	-	[3]
***Pseudorhypophila marina***	CBS 155.77^T^	MK926851	MK926851	MK876813	-	[3]
	CBS 698.96^T^	MK926853	MK926853	MK876815	-	[3]
***Pseudorhypophila pilifera***	CBS 413.73^T^	MK926852	MK926852	MK876814	-	[3]
***Pseudorhypophila mangenotii***	CBS 419.67^T^	KP981444	MT784143	KP981627	KP981571	[4]
*Pseudoschizothecium atropurpureum*	SMH 3073	AY780057	-	AY780160	AY780100	[1]
*Rinaldiella pentagonospora*	CBS 132344^T^	KP981442	MH866007	KP981625	KP981570	[4,25]
*Rhypophila cochleariformis*	CBS 249.71	AY999098	AY999123	-	-	[18]
*Rhypophila decipiens*	CBS 258.69	AY780073	KX171946	AY780187	AY780130	[1], Miller [unpubl. data]
*Rhypophila myriaspora*	TNM F17211	-	EF197083	-	-	[26]
*Rhypophila pleiospora*	TNM F16889	-	EF197084	-	-	[26]
*Schizothecium inaequale*	CBS 356.49^T^	MK926846	MK926846	MK876808	-	[3]
*Schizothecium selenosporum*	CBS 109403^T^	MK926849	MK926849	MK876811	-	[3]
Sordaria araneosa*	F-116,361	-	FJ175160	-	-	[8]
*Triangularia allahabadensis*	CBS 724.68^T^	MK926865	MK926865	MK876827	-	[3]
*Triangularia anserina*	CBS 433.50	MK926864	MK926864	-	MK876826	[3]
*Triangularia arizonensis*	Kruys 724	KF557669	-	KF557707	-	[2]
*Triangularia backusii*	CBS 539.89^IsoT^	MK926866	MK926866	MK876828	-	[3]
	FMR 12439	KP981423	MT784138	KP981605	KP981550	[4]
*Triangularia bambusae*	CBS 352.33^T^	MK926868	MK926868	MK876830	-	[3]
*Triangularia batistae*	CBS 381.68^T^	KP981443	MT784140	KP981626	KP981577	[4]
*Triangularia longicaudata*	CBS 252.57^T^	MK926871	MK926871	MK876833	-	[3]
*Triangularia pauciseta*	CBS 451.62	MK926870	MK926870	-	MK876832	[3]
*Triangularia phialophoroides*	CBS 301.90^T^	MK926871	MK926871	-	MK876833	[3]
*Triangularia setosa*	FMR 12787	KP981441	MT784144	KP981624	KP981569	[4]
*Triangularia striata*	SMH 3431	-	AY780065	AY780108	AY780169	[1]
	SMH 4036	KX348038	AY780066	-	-	[1], Miller [unpubl. data]
*Triangularia tetraspora*	FMR 5770	AY999130	AY999108	-	-	[27]
*Triangularia verruculosa*	CBS 148.77	MK926874	MK926874	MK876836	-	[3]
Zopfiella attenuata*	CBS 266.77^T^	KP981445	MH861060	KP981628	KP981572	[4,25]
Zopfiella tardifaciens*	CBS 670.82^T^	MK926855	MK926855	MK876817	-	[3]

CBS: Westerdijk Fungal Biodiversity Institute, Utrecht, the Netherlands; FMR: Facultat de Medicina, Reus, Spain; IMI: International Mycological Institute, CABI-Bioscience, Egham, UK; NBRC: Biological Resource Center, Chiba, Japan; SANK: Research laboratories of the Daiichi Sanko Pharmaceutical Co., Ltd., Tokyo, Japan; TNM: Herbarium of National Museum of Natural Science, Taiwan; TRTC: Royal Ontario Museum, Toronto, Canada; UAMH: UAMH Center for Global Microfungal Biodiversity, University of Toronto, Canada; Francoise Candoussau, JF, Kruys, Lundqvist, MJR, SMH: personal collections of Francoise Candoussau, Jacques Fournier, Åsa Kruys, Nils Lundqvist, Michael J. Richardson and Sabine M. Huhndorf, respectively; n/a: not available. ^ET, IsoT^ and ^T^ indicates ex-epitype, ex-isotype and ex-type strains, respectively. * Taxa with generic names applied in the broad sense (*sensu lato*), not necessarily reflecting molecular phylogenetic relationships.

**Table 2:** NMR data of metabolites **1**–**4** in DMSO-*d*_6_ (^1^H 500 MHz, ^13^C 125 MHz).

	1		2		3		4	
	*δ*_C_, Type	*δ*_H_, Multiplicity	*δ*_C_, Type	*δ*_H_, Multiplicity	*δ*_C_, Type	*δ*_H_, Multiplicity	*δ*_C_, Type	*δ*_H_, Multiplicity
1	55.9, CH_2_	4.64, s	54.2, CH_2_	4.54, s	55.9, CH_2_	4.64, s	54.2, CH_2_	4.54, s
2	126.6, C		124.7, C		126.6, C		124.7, C	
3	151.3, C		155.6, C	OH: 9.33, br s	151.3, C		155.6, C	
4	119.2, C		114.0, CH	6.68, br d (7.9)	119.2, C		113.9, C	6.69, dd (7.9,1.0)
5	128.2, CH	7.21, d (8.4)	127.9, CH	7.02, t (7.9)	128.2, CH	7.22, d (8.4)	128.0, CH	7.02, t (7.9)
6	117.9, CH	6.92, d (8.4)	116.5, CH	6.89, m	118.0, CH	6.97, d (8.4)	116.5, CH	6.92, d (7.9)
7	136.7, C		138.4, C		136.8, C		138.4, C	
8	126.2, CH	6.79, d (15.8)	127.4, CH	6.88, m	126.1, CH	6.80, d (15.8)	127.3, CH	6.89, d (15.8)
9	133.8, CH	6.13, dd (15.8, 5.8)	132.6, CH	6.11, dd (16.0, 6.1)	134.3, CH	6.19, dd (15.8, 6.3)	133.0, CH	6.16, dd (15.8, 6.3)
10	75.0, CH	3.99, br dd (5.8, 5.0)	75.2, CH	3.99, pseudo q (5.0)	75.0, CH	3.91, ddd (6.3,5.2,5.0)	75.2, CH	3.92, ddd (6.3,5.2,5.0)
	OH	4.87, br s		4.81, br d (4.9)	OH	4.84, d (5.2)	OH	4.79, d (5.2)
11	74.8, CH	3.87, br dd (6.2, 5.0)	74.9, CH	3.87, pseudo q (4.7)	73.7, CH	3.34, m	73.8, CH	3.34, m
	OH	4.71, br s		4.68, br d (4.9)	OH	4.42, d (5.8)	OH	4.38, d (5.8)
12	130.8, CH	5.51, dd (15.6, 6.2)	130.9, CH_2_	5.51, dt (15.6, 6.2)	32.6, CH_2_	1.50, m 1.26, m	32.6, CH_2_	1.50, m 1.26, m
13	130.7, CH	5.58, dt (15.6, 6.4)	130.6, CH_2_	5.58, dt (15.6, 6.2)	25.4, CH_2_	1.45, m 1.25, m	25.4, CH_2_	1.45, m 1.25, m
14	31.7, CH_2_	1.98, pseudo q (6.9)	31.8, CH_2_	1.98, pseudo q (6.8)	29.2, CH_2_	1.25, m	29.2, CH_2_	1.25, m
15	28.5, CH_2_	1.32, m	28.5, CH_2_	1.32, m	28.8, CH_2_	1.25, m	28.8, CH_2_	1.25, m
16	30.7, CH_2_	1.24, m	30.7, CH_2_	1.25, m	31.3, CH_2_	1.24, m	31.3, CH_2_	1.24, m
17	21.9, CH_2_	1.24, m	22.0, CH_2_	1.25, m	22.1, CH_2_	1.26, m	22.1, CH_2_	1.26, m
18	13.9, CH_3_	0.83, t (6.9)	13.9, CH_3_	0.84, t (6.9)	14.0, CH_3_	0.85, t (6.9)	14.0, CH_3_	0.85, t (6.9)

**Table 3:** Minimum inhibitory concentration (MIC, µg/mL) of **1**–**9** against fungi and bacteria.

Test Organism	1	2	3	4	5	6	7	8	9	Positive Control
*Candida albicans*	–	–	–	–	–	–	33.3	–	66.7	4.20 ^N^
*Schizosaccharomyces pombe*	–	–	–	–	–	–	–	–	–	4.20 ^N^
*Wickerhamomyces anomalus*	–	–	–	–	–	–	–	–	–	4.20 ^N^
*Rhodotorula glutinis*	–	–	66.7	–	–	–	–	–	–	1.00 ^N^
*Mucor hiemalis*	66.7	–	66.7	–	–	–	–	–	16.7	2.10 ^N^
*Mycobacterium smegmatis*	–	–	–	–	–	–	–	–	–	1.70 ^K^
*Bacillus subtilis*	33.3	–	33.3	66.7	–	–	–	–	–	8.30 ^O^
*Staphylococcus aureus*	66.7	–	33.3	66.7	–	–	–	–	–	0.83 ^O^
*Chromobacterium violaceum*	–	–	–	–	–	–	–	–	–	0.83 ^O^
*Escherichia coli*	–	–	–	–	–	–	–	–	–	1.70 ^O^
*Pseudomonas aeruginosa*	–	–	–	–	–	–	–	–	–	0.42 ^G^

^G^ gentamicin, ^K^ kanamycin, ^O^ oxytetracycline, ^N^ nystatin, –: no inhibition observed under test conditions.

**Table 4:** Cytotoxicity of **1**–**9** against mammalian cell lines [half maximal inhibitory concentrations (IC_50_): µM].

Cell Lines	1	2	3	4	5	6	7	8	9	Epothilone B
KB 3.1	15.6	–	23.0	23.7	–	–	–	–	–	0.00003
L929	52.9	–	70.4	45.5	–	–	–	–	–	0.00051

–: no inhibition observed under test conditions.

## Data Availability

The DNA sequences are deposited in GenBank (https://www.ncbi.nlm.nih.gov/genbank/) and all other relevant data are included in the Supplementary Information.

## Supplementary Materials

The following are available online at https://www.mdpi.com/2309-608X/7/3/181/s1, Figure S1: ^1^H NMR spectrum (500 MHz, DMSO-*d*_6_) of zopfinol (**1**), Figure S2: ^13^C NMR spectrum (125 MHz, DMSO-*d*_6_) of zopfinol (**1**), Figure S3: COSY NMR spectrum (500 MHz, DMSO-*d*_6_) of zopfinol (**1**), Figure S4: ROESY NMR spectrum (500 MHz, DMSO-*d*_6_) of zopfinol (**1**), Figure S5: HSQC NMR spectrum (500 MHz, DMSO-*d*_6_) of zopfinol (**1**), Figure S6: HMBC NMR spectrum (500 MHz, DMSO-*d*_6_) of zopfinol (**1**), Figure S7: ^1^H NMR spectrum (500 MHz, DMSO-*d*_6_) of zopfinol B (**2**), Figure S8: ^13^C NMR spectrum (125 MHz, DMSO-*d*_6_) of zopfinol B (**2**), Figure S9: COSY NMR spectrum (500 MHz, DMSO-*d*_6_) of zopfinol B (**2**), Figure S10: NOESY NMR spectrum (500 MHz, DMSO-*d*_6_) of zopfinol B (**2**), Figure S11: HSQC NMR spectrum (500 MHz, DMSO-*d*_6_) of zopfinol B (**2**), Figure S12: HMBC NMR spectrum (500 MHz, DMSO-*d*_6_) of zopfinol B (**2**), Figure S13: ^1^H NMR spectrum (500 MHz, DMSO-*d*_6_) of zopfinol C (**3**), Figure S14: ^13^C NMR spectrum (125 MHz, DMSO-*d*_6_) of zopfinol C (**3**), Figure S15: COSY NMR spectrum (500 MHz, DMSO-*d*_6_) of zopfinol C (**3**), Figure S16: ROESY NMR spectrum (500 MHz, DMSO-*d*_6_) of zopfinol C (**3**), Figure S17: HSQC NMR spectrum (500 MHz, DMSO-*d*_6_) of zopfinol C (**3**), Figure S18: HMBC NMR spectrum (500 MHz, DMSO-*d*_6_) of zopfinol C (**3**), Figure S19: ^1^H NMR spectrum (500 MHz, DMSO-*d*_6_) of zopfinol D (**4**), Figure S20: ^13^C NMR spectrum (125 MHz, DMSO-*d*_6_) of zopfinol D (**4**), Figure S21: COSY NMR spectrum (500 MHz, DMSO-*d*_6_) of zopfinol D (**4**), Figure S22: ROESY NMR spectrum (500 MHz, DMSO-*d*_6_) of zopfinol D (**4**), Figure S23: HSQC NMR spectrum (500 MHz, DMSO-*d*_6_) of zopfinol D (**4**), Figure S24: HMBC NMR spectrum (500 MHz, DMSO-*d*_6_) of zopfinol D (**4**), Figure S25: HSQC NMR spectrum (700 MHz, pyridin-d5) of zopfinol A-S-MTPA ester, Figure S26: HSQC NMR spectrum (700 MHz, pyridin-*d*_5_) of zopfinol A-*R*-MTPA ester.
