# Supplementary material for: Three New Derivatives of Zopfinol from Pseudorhypophila Mangenotii gen. et comb. nov
Source: J Fungi (Basel). 2021 Mar 3;7(3):181. doi: 10.3390/jof7030181 (PMC8000789; doi:10.3390/jof7030181)
Supplement: Supplementary file 1 [file jof-07-00181-s001.pdf]

Supporting Information for

Three new derivatives of Zopfinol from *Pseudorhypophila mangenotii* gen. et comb. nov.

Karen Harms [1,2], Andrea Milic [1], Alberto M. Stschigel [3], Marc Stadler [1,2], Frank Surup [1,2,*], Yasmina Marin-Felix [1,*]

[1] Department Microbial Drugs, Helmholtz Centre for Infection Research GmbH and German Centre for Infection Research (DZIF), Partner Site Hannover-Braunschweig, 38124 Braunschweig, Germany; andrea.milic@student.uni-tuebingen.de (A.M.); Yasmina.MarinFelix@helmholtz-hzi.de (Y.M.F.)

[2] Institute of Microbiology, Technische Universität Braunschweig, Inhoffenstraße 7, 38124 Braunschweig, Germany; Karen.Harms@helmholtz-hzi.de (K.H.); Frank.Surup@helmholtz-hzi.de (F.S.); Marc.Stadler@helmholtz-hzi.de (M.S.)

[3] Mycology Unit, Medical School and IISPV, Universitat Rovira i Virgili, C/ Sant Llorenç 21, 43201 Reus, Tarragona, Spain; albertomiguel.stchigel@urv.cat (A.M.S.)

* Correspondences: Yasmina.MarinFelix@helmholtz-hzi.de and Frank.Surup@helmholtz-hzi.de

Contents

Figure S1. ^{1}H NMR spectrum (500 MHz, DMSO-d_6) of zopfinol (**1**) 4

Figure S2. ^{13}C NMR spectrum (125 MHz, DMSO-d_6) of zopfinol (**1**)....................5

Figure S3. COSY NMR spectrum (500 MHz, DMSO-d_6) of zopfinol (**1**) 6

Figure S4. ROESY NMR spectrum (500 MHz, DMSO-d_6) of zopfinol (**1**) 7

Figure S5. HSQC NMR spectrum (500 MHz, DMSO-d_6) of zopfinol (**1**)....................8

Figure S6. HMBC NMR spectrum (500 MHz, DMSO-d_6) of zopfinol (**1**)....................9

Figure S7. ^{1}H NMR spectrum (500 MHz, DMSO-d_6) of zopfinol B (**2**) 10

Figure S8. ^{13}C NMR spectrum (125 MHz, DMSO-d_6) of zopfinol B (**2**) 11

Figure S9. COSY NMR spectrum (500 MHz, DMSO-d_6) of zopfinol B (**2**) 12

Figure S10. NOESY NMR spectrum (500 MHz, DMSO-d_6) of zopfinol B (**2**)....................13

Figure S11. HSQC NMR spectrum (500 MHz, DMSO-d_6) of zopfinol B (**2**) 14

Figure S12. HMBC NMR spectrum (500 MHz, DMSO-d_6) of zopfinol B (**2**) 15

Figure S13. ^{1}H NMR spectrum (500 MHz, DMSO-d_6) of zopfinol C (**3**)....................16

Figure S14. ^{13}C NMR spectrum (125 MHz, DMSO-d_6) of zopfinol C (**3**)....................17

Figure S15. COSY NMR spectrum (500 MHz, DMSO-d_6) of zopfinol C (**3**) 18

Figure S16. ROESY NMR spectrum (500 MHz, DMSO-d_6) of zopfinol C (**3**) 19

Figure S17. HSQC NMR spectrum (500 MHz, DMSO-d_6) of zopfinol C (**3**)....................20

Figure S18. HMBC NMR spectrum (500 MHz, DMSO-d_6) of zopfinol C (**3**)....................21

Figure S19. ^{1}H NMR spectrum (500 MHz, DMSO-d_6) of zopfinol D (**4**) 22

Figure S20. ^{13}C NMR spectrum (125 MHz, DMSO-d_6) of zopfinol D (**4**)....23

Figure S21. COSY NMR spectrum (500 MHz, DMSO-d_6) of zopfinol D (**4**)....24

Figure S22. ROESY NMR spectrum (500 MHz, DMSO-d_6) of zopfinol D (**4**)....25

Figure S23. HSQC NMR spectrum (500 MHz, DMSO-d_6) of zopfinol D (**4**)....26

Figure S24. HMBC NMR spectrum (500 MHz, DMSO-d_6) of zopfinol D (**4**)....27

Figure S25. HSQC NMR spectrum (700 MHz, pyridin-d_5) of zopfinol A-*S*-MTPA ester....28

Figure S26. HSQC NMR spectrum (700 MHz, pyridin-d_5) of zopfinol A-*R*-MTPA ester....29

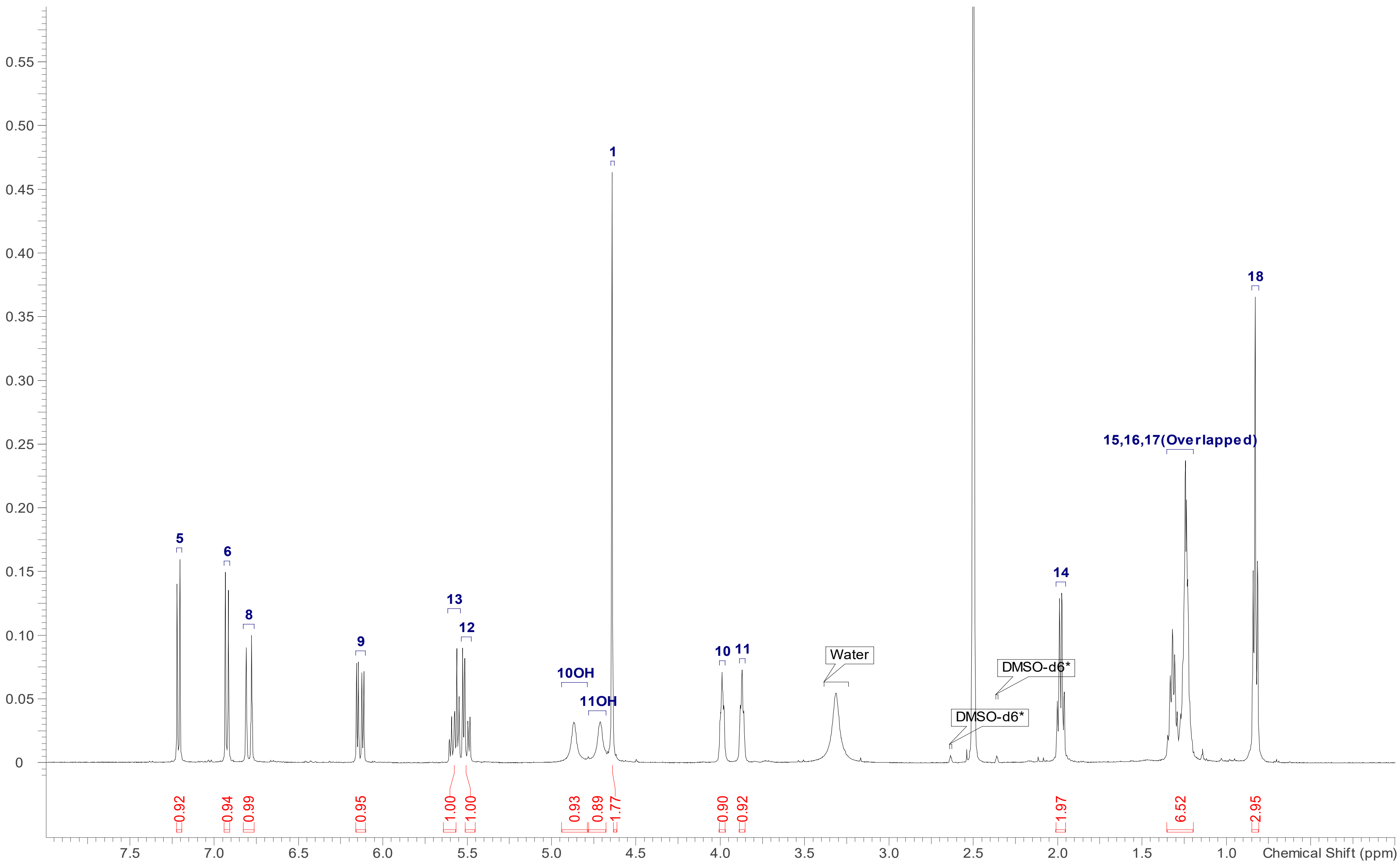

Figure S1. ^1H NMR spectrum (500 MHz, DMSO-d_6) of zopfinol (**1**).

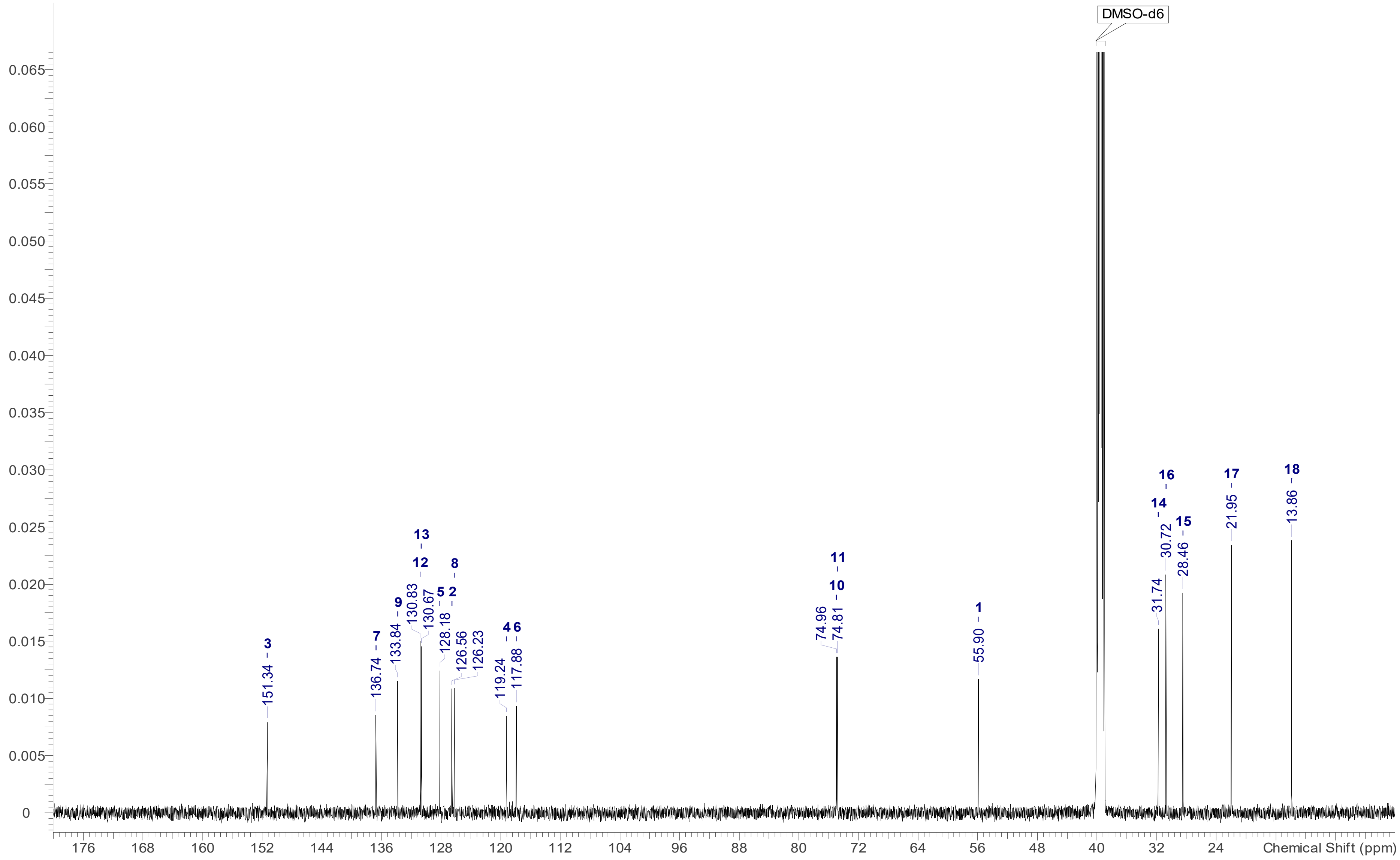

Figure S2. ^{13}C NMR spectrum (125 MHz, DMSO-d_6) of zopfinol (**1**).

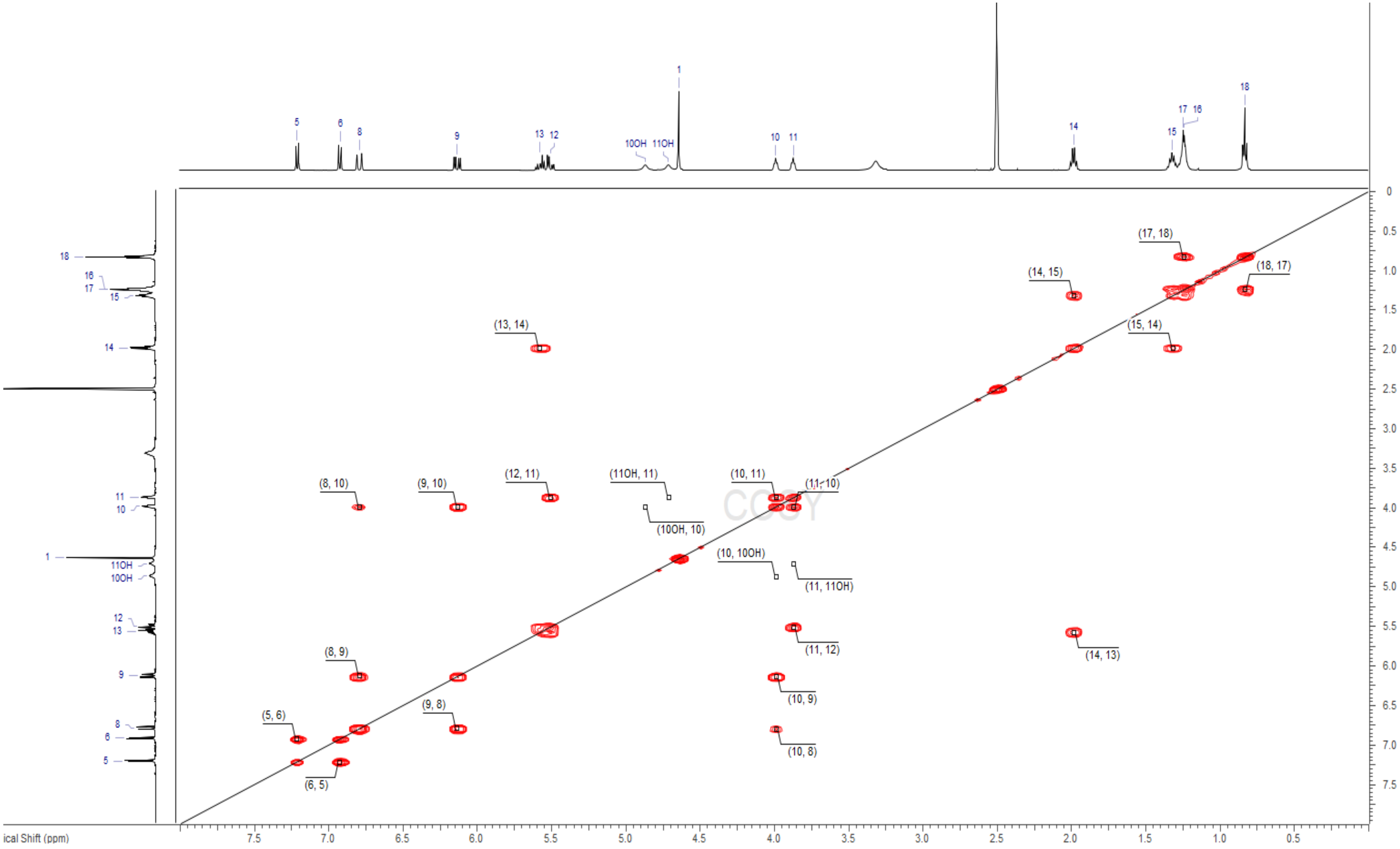

Figure S3. COSY NMR spectrum (500 MHz, DMSO-d_6) of zopfinol (**1**).

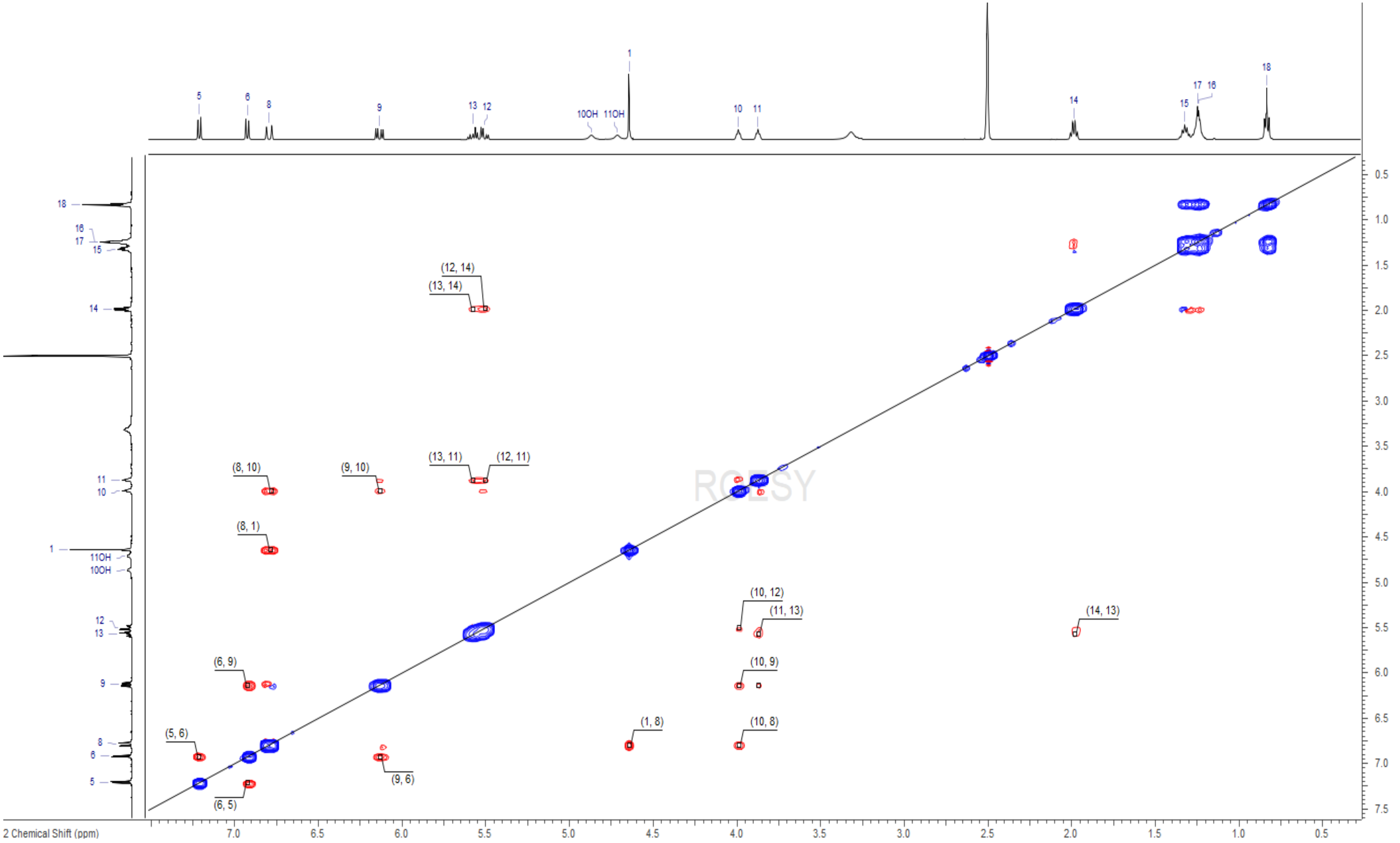

Figure S4. ROESY NMR spectrum (500 MHz, DMSO-d_6) of zopfinol (**1**).

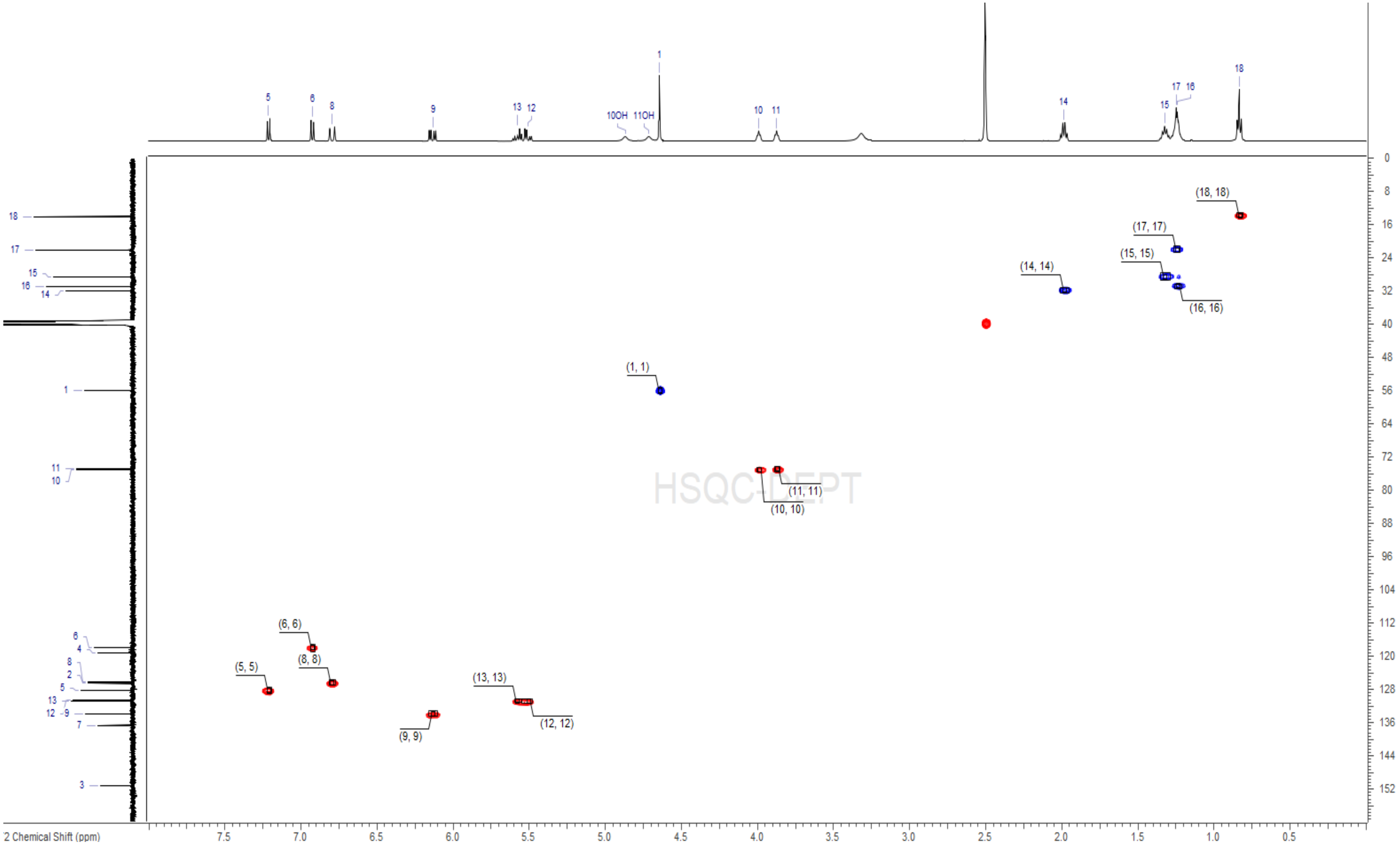

Figure S5. HSQC NMR spectrum (500 MHz, DMSO-d_6) of zopfinol (**1**).

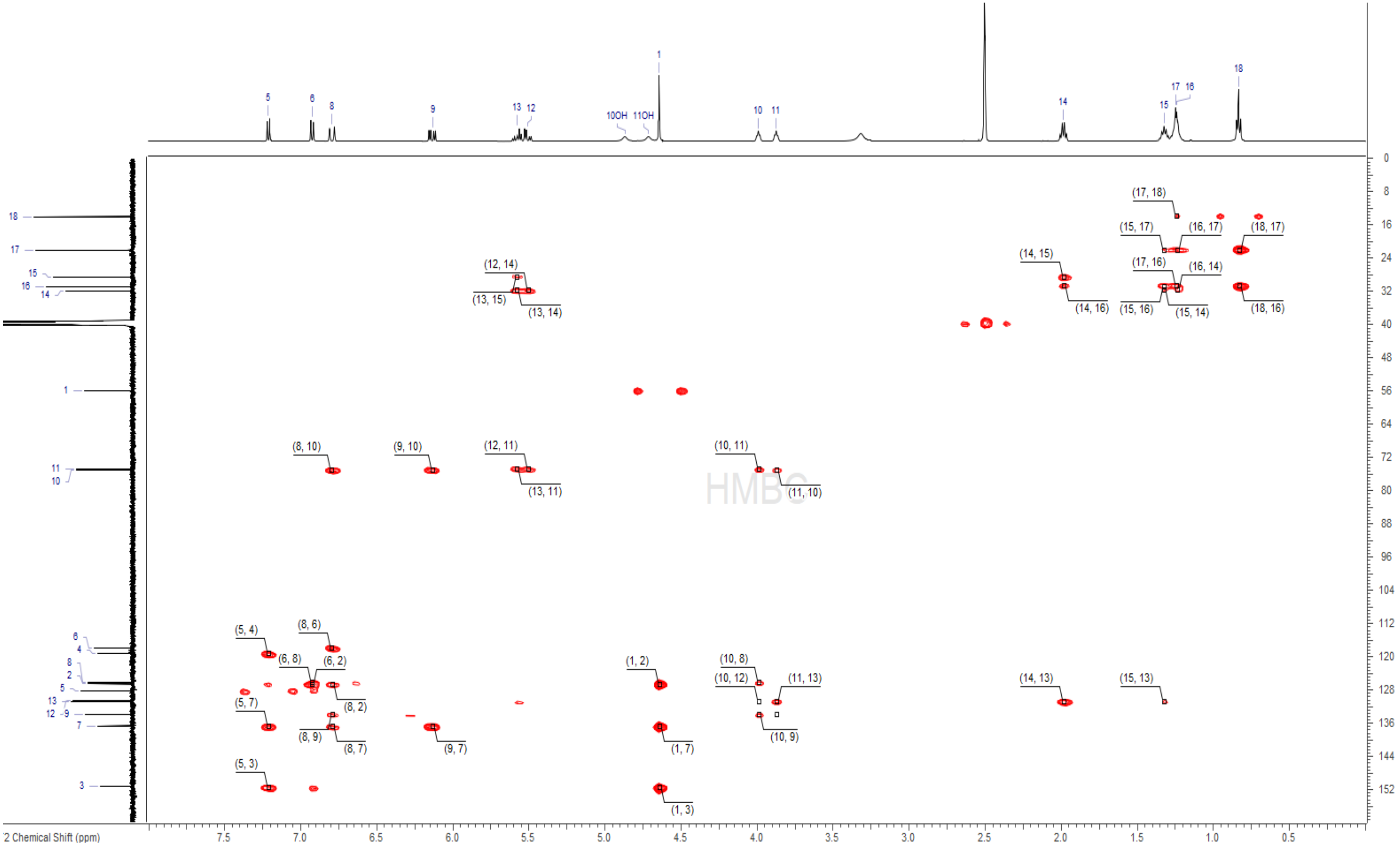

Figure S6. HMBC NMR spectrum (500 MHz, DMSO-d_6) of zopfinol (**1**).

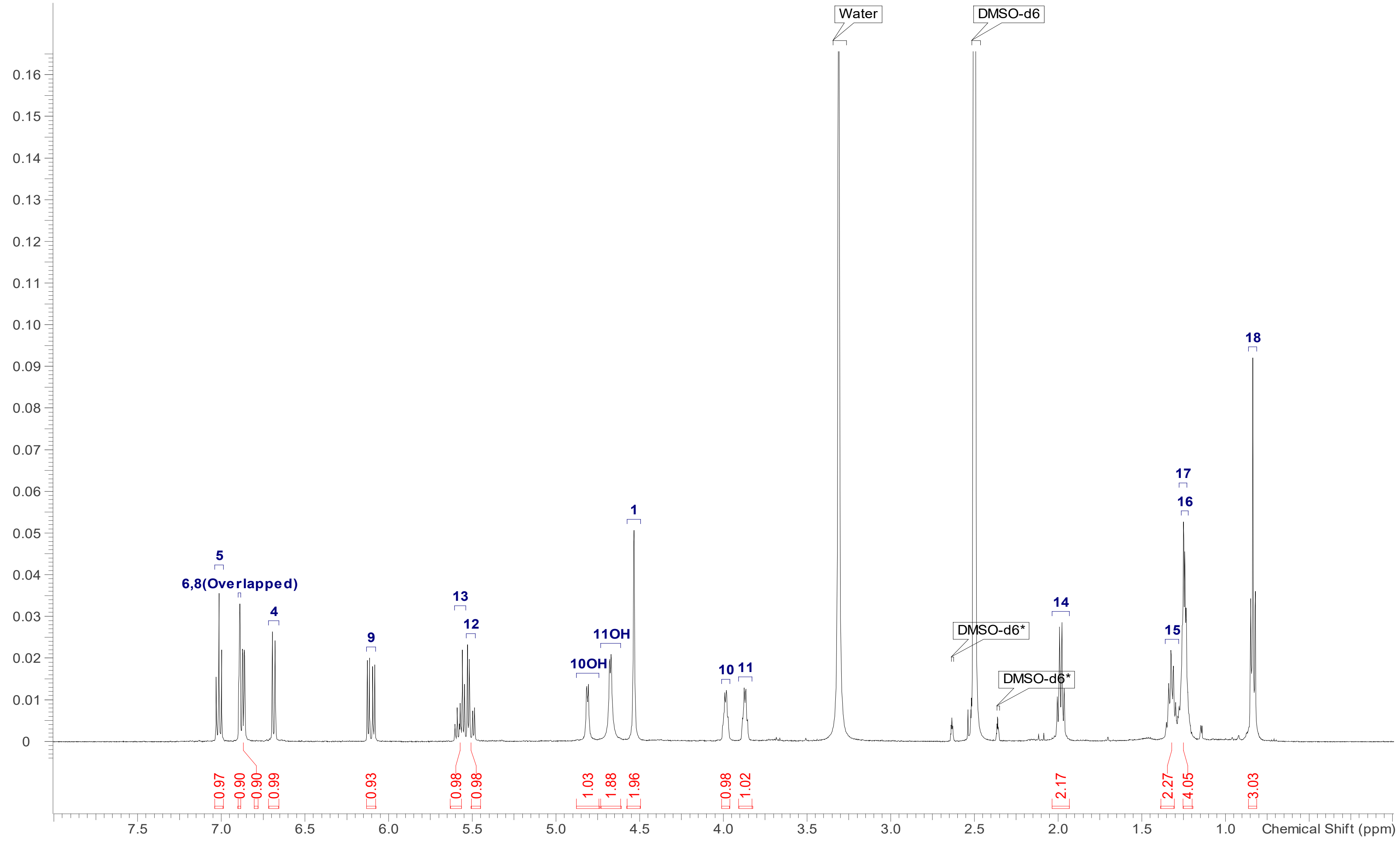

Figure S7. ^{1}H NMR spectrum (500 MHz, DMSO-d_6) of zopfinol B (**2**).

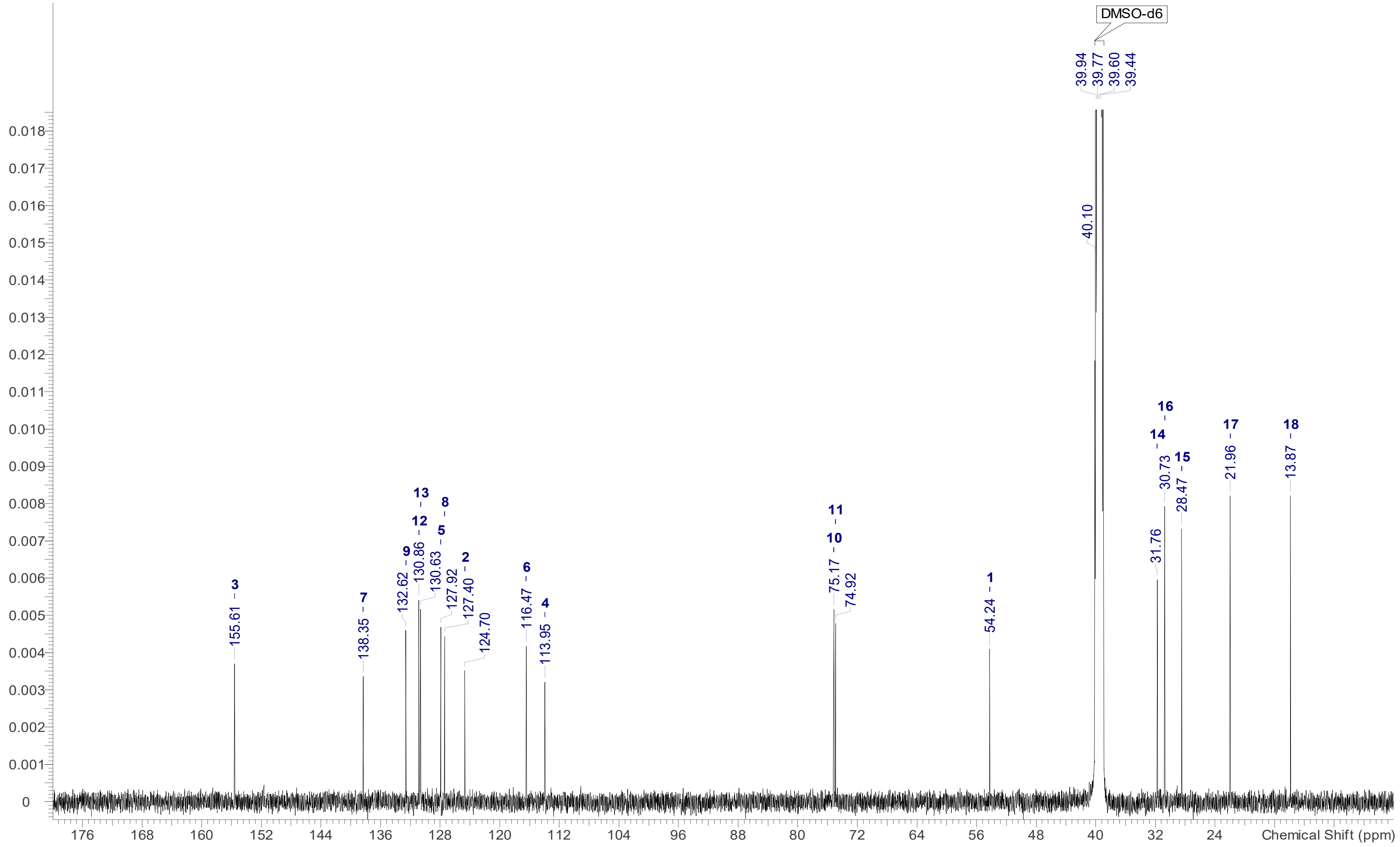

Figure S8. ^{13}C NMR spectrum (125 MHz, DMSO-d_6) of zopfinol B (**2**).

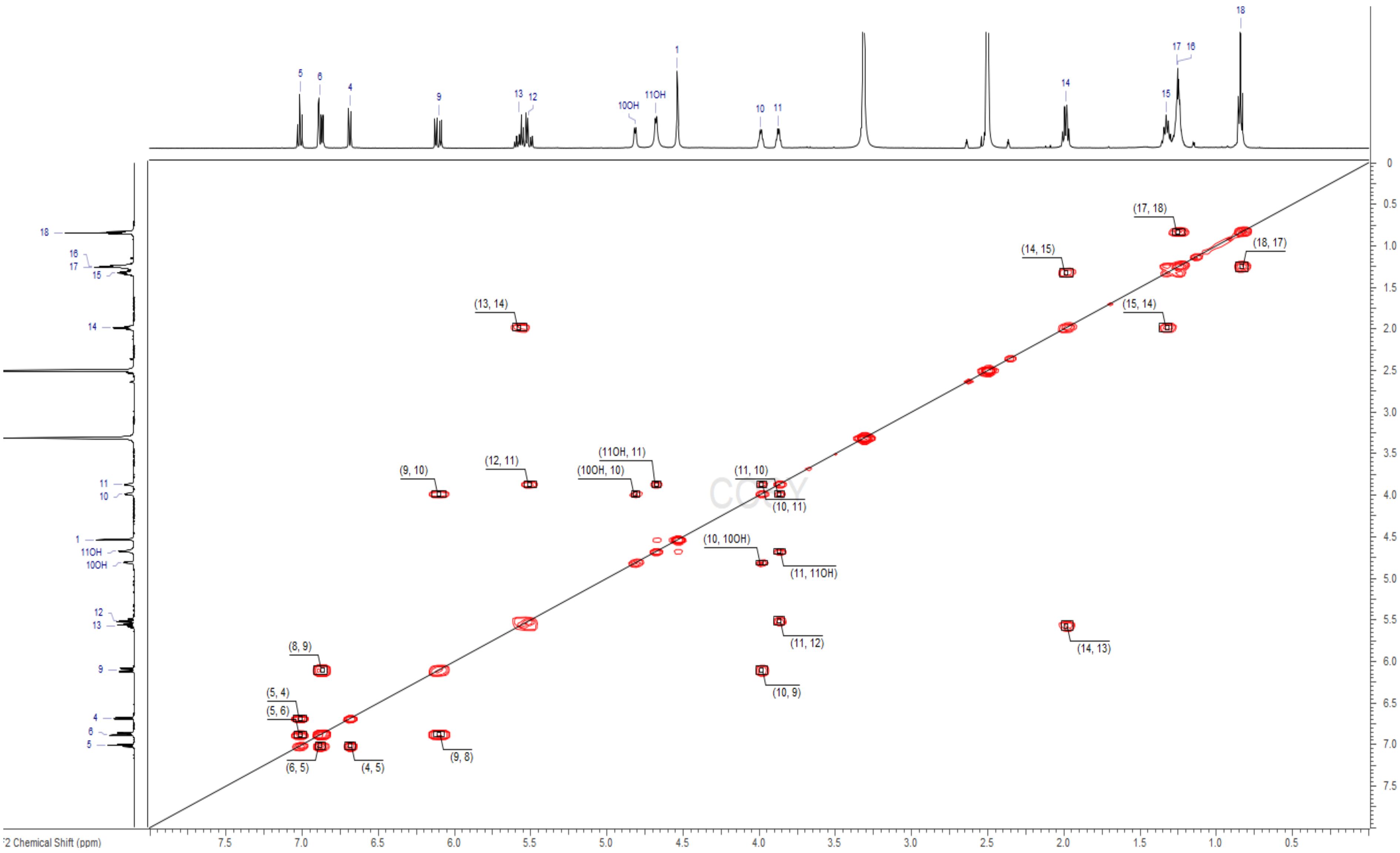

Figure S9. COSY NMR spectrum (500 MHz, DMSO-d_6) of zopfinol B (**2**).

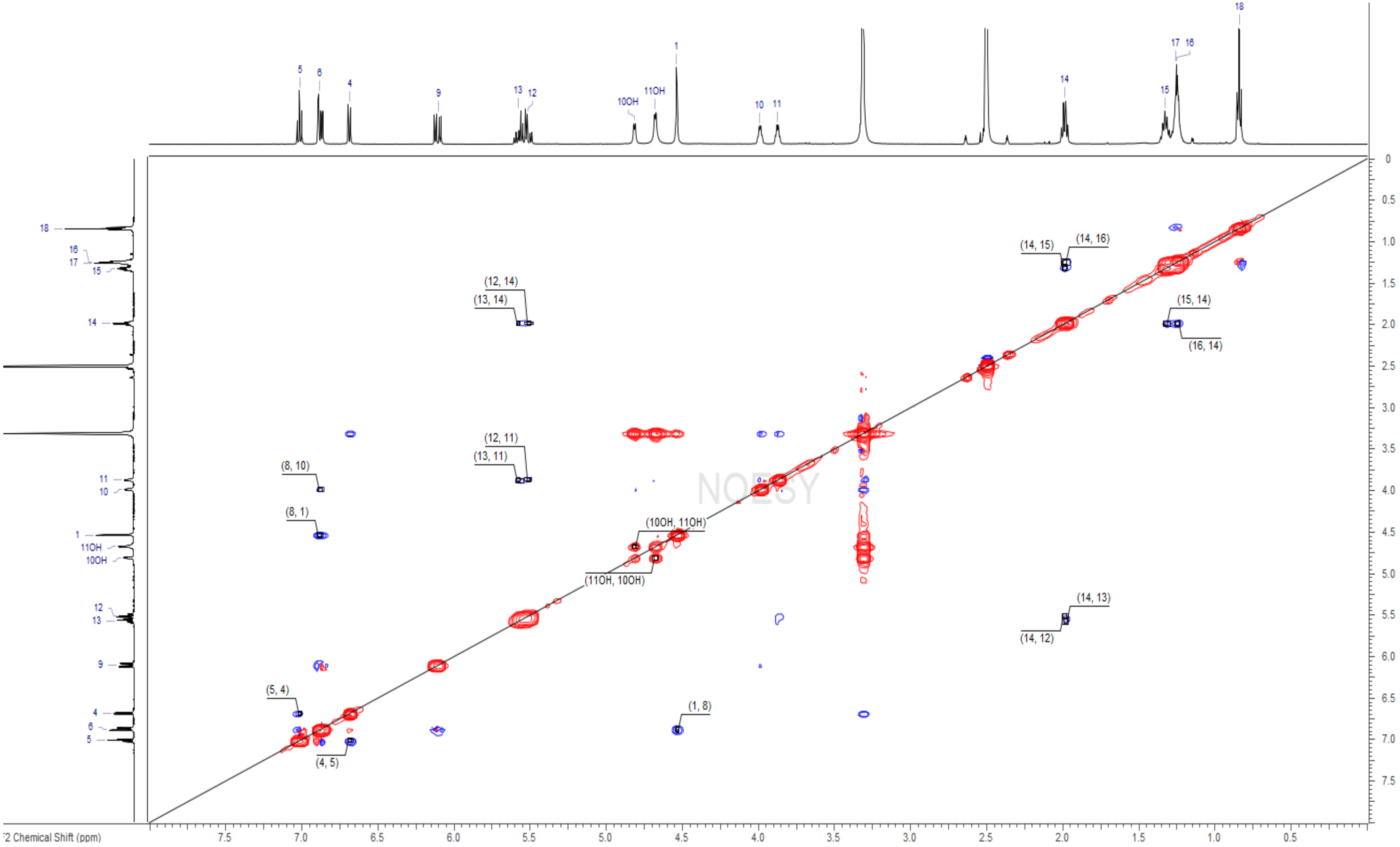

Figure S10. NOESY NMR spectrum (500 MHz, DMSO-d_6) of zopfinol B (**2**).

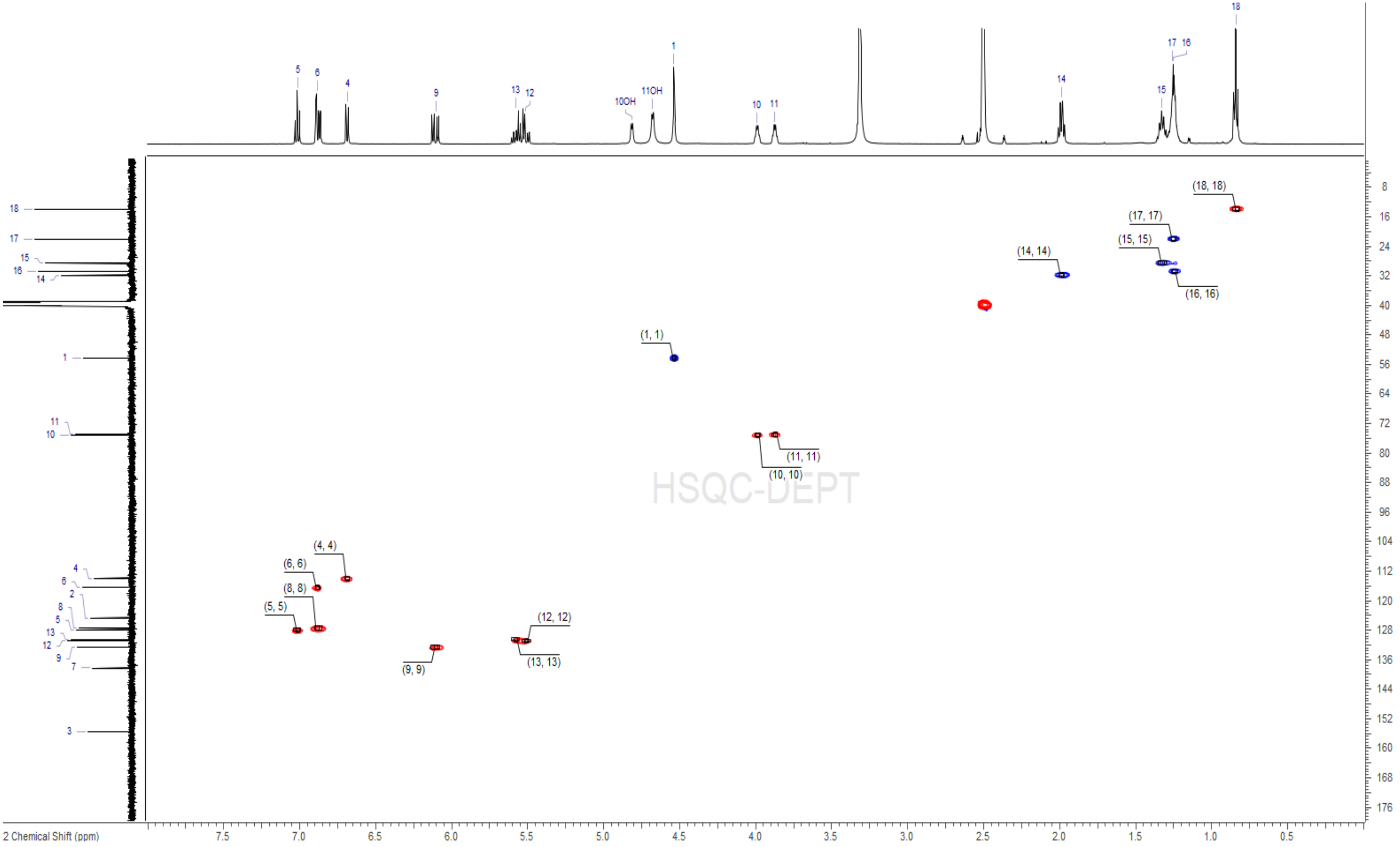

Figure S11. HSQC NMR spectrum (500 MHz, DMSO-d_6) of zopfinol B (**2**).

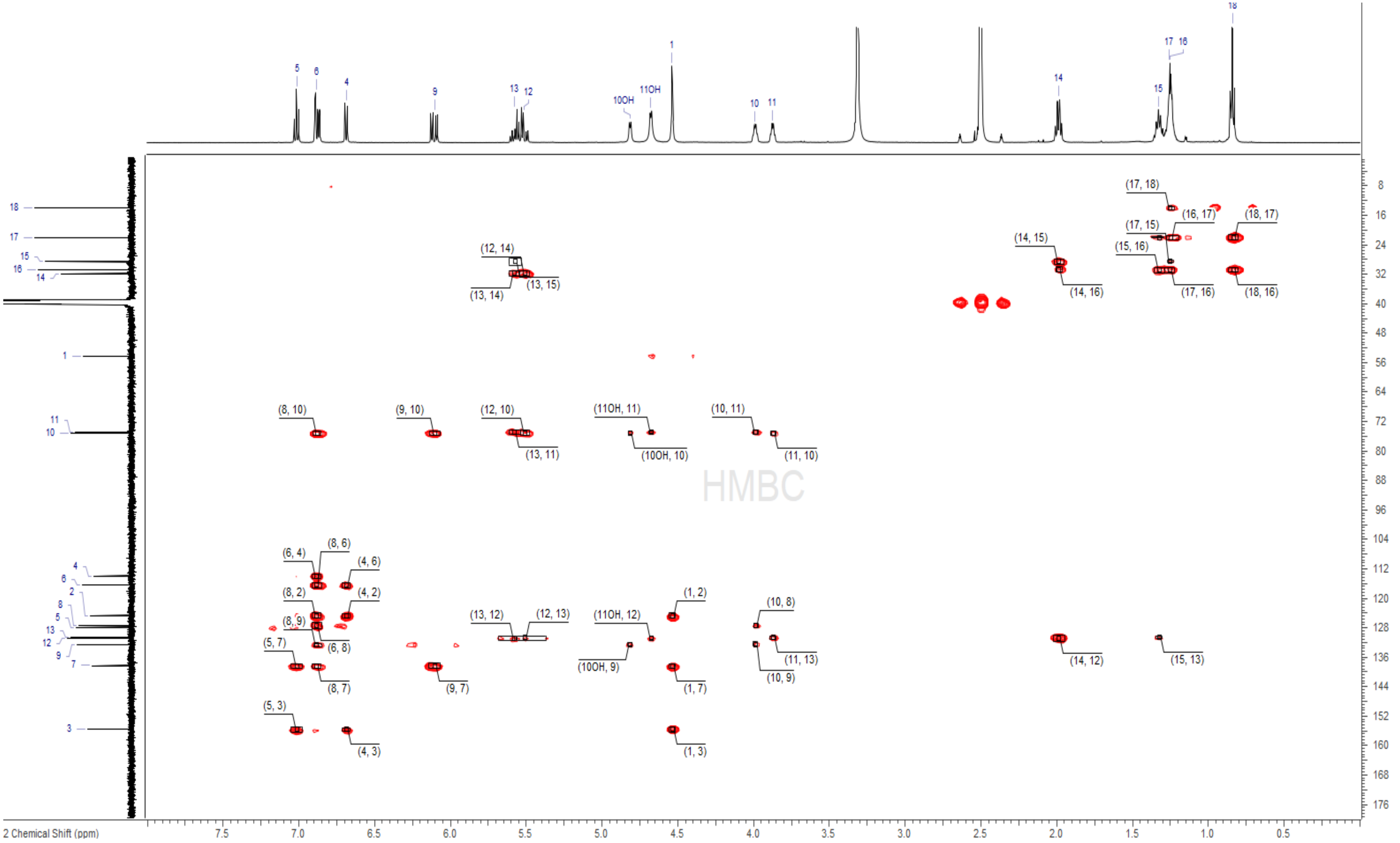

Figure S12. HMBC NMR spectrum (500 MHz, DMSO-d_6) of zopfinol B (**2**).

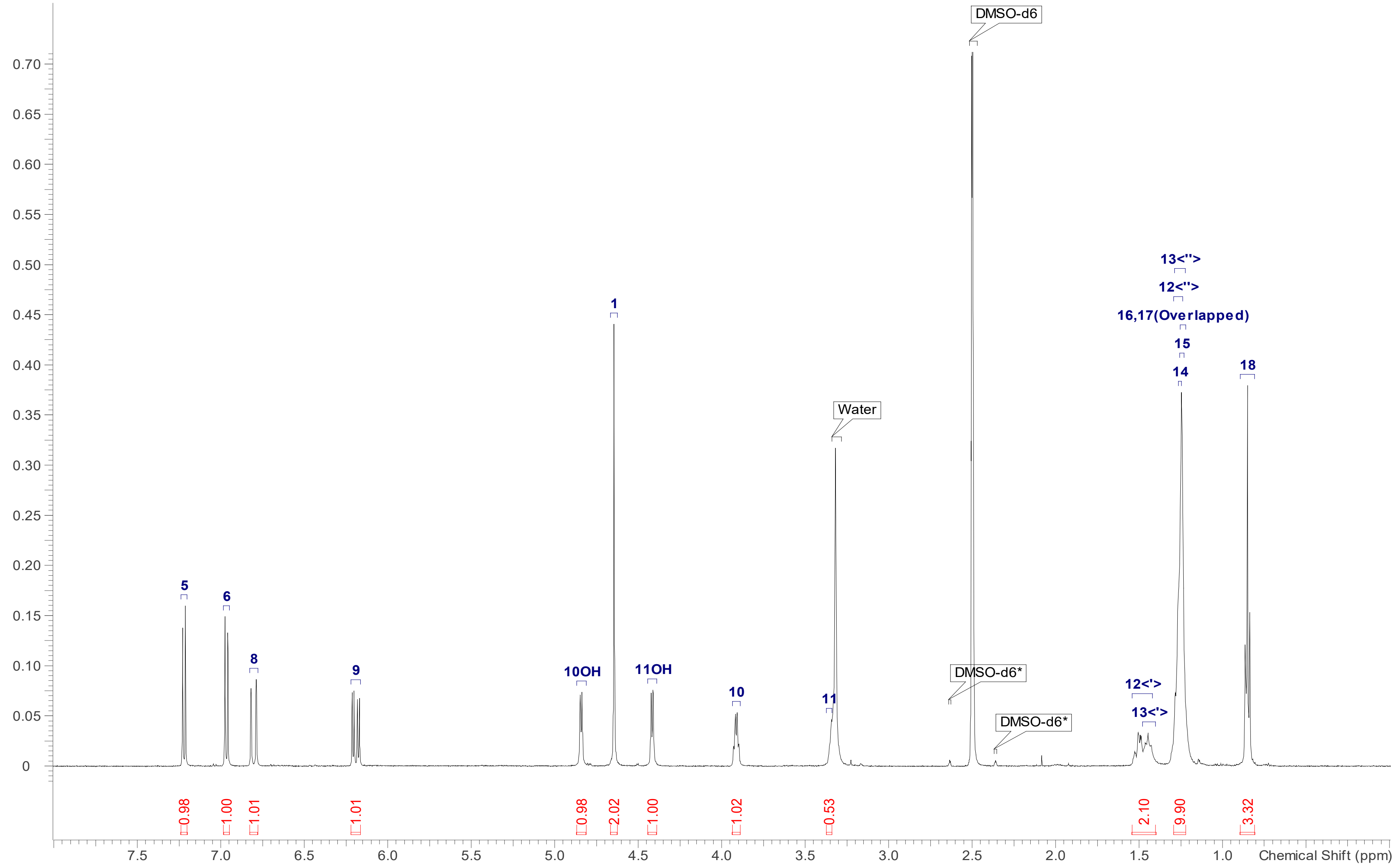

Figure S13. ^{1}H NMR spectrum (500 MHz, DMSO-d_6) of zopfinol C (**3**).

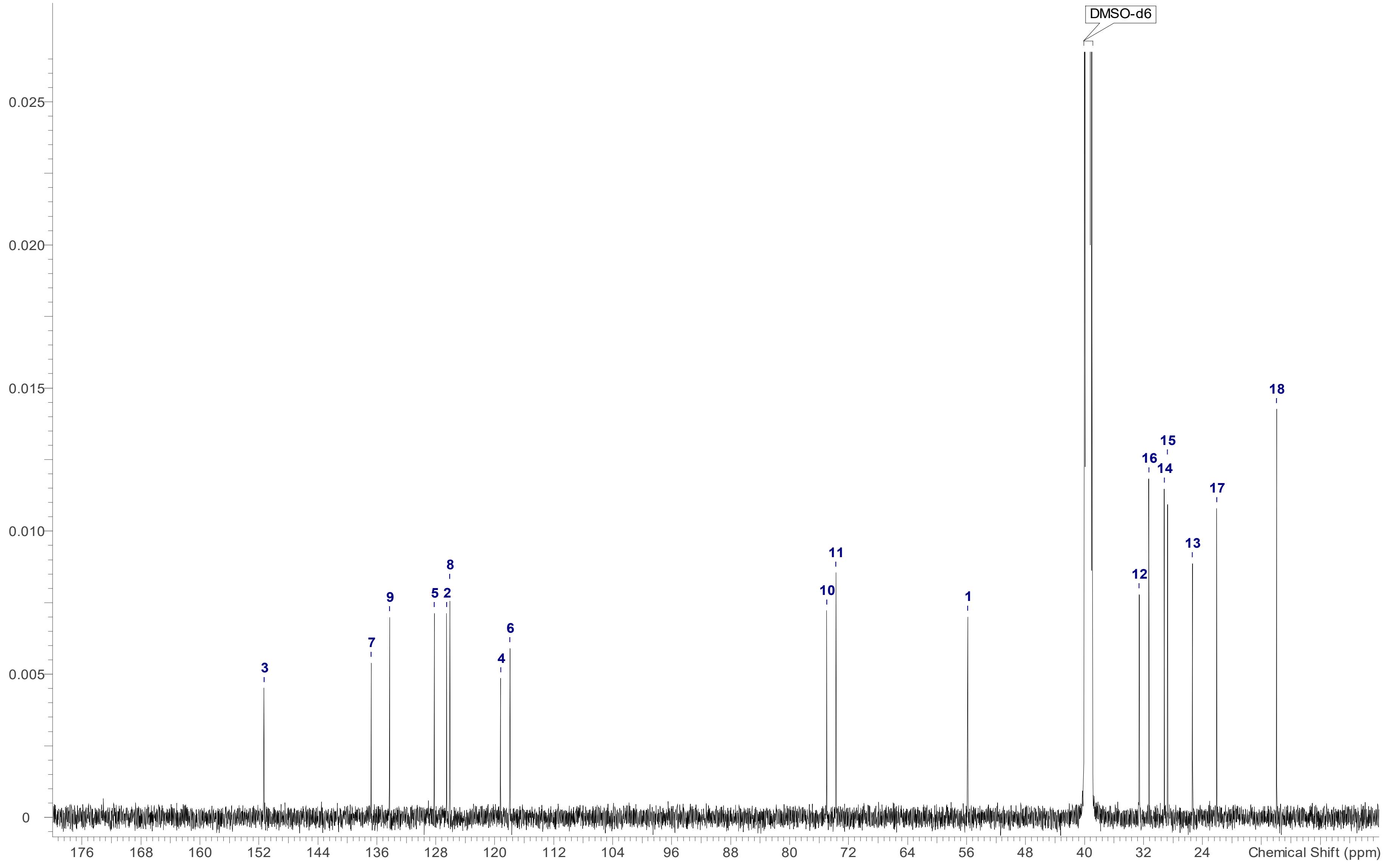

Figure S14. ^{13}C NMR spectrum (125 MHz, DMSO-d_6) of zopfinol C (**3**).

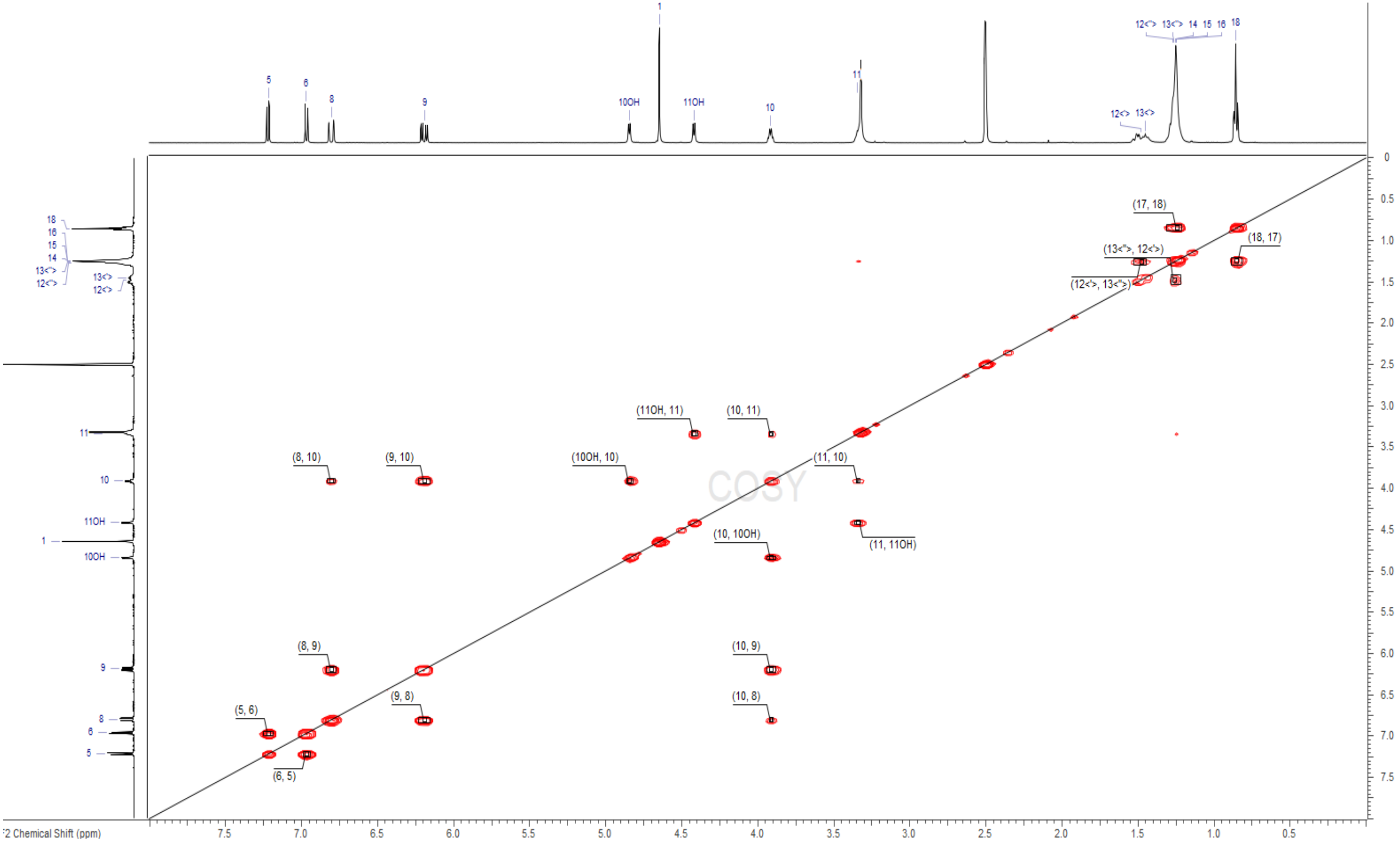

Figure S15. COSY NMR spectrum (500 MHz, DMSO-d_6) of zopfinol C (**3**).

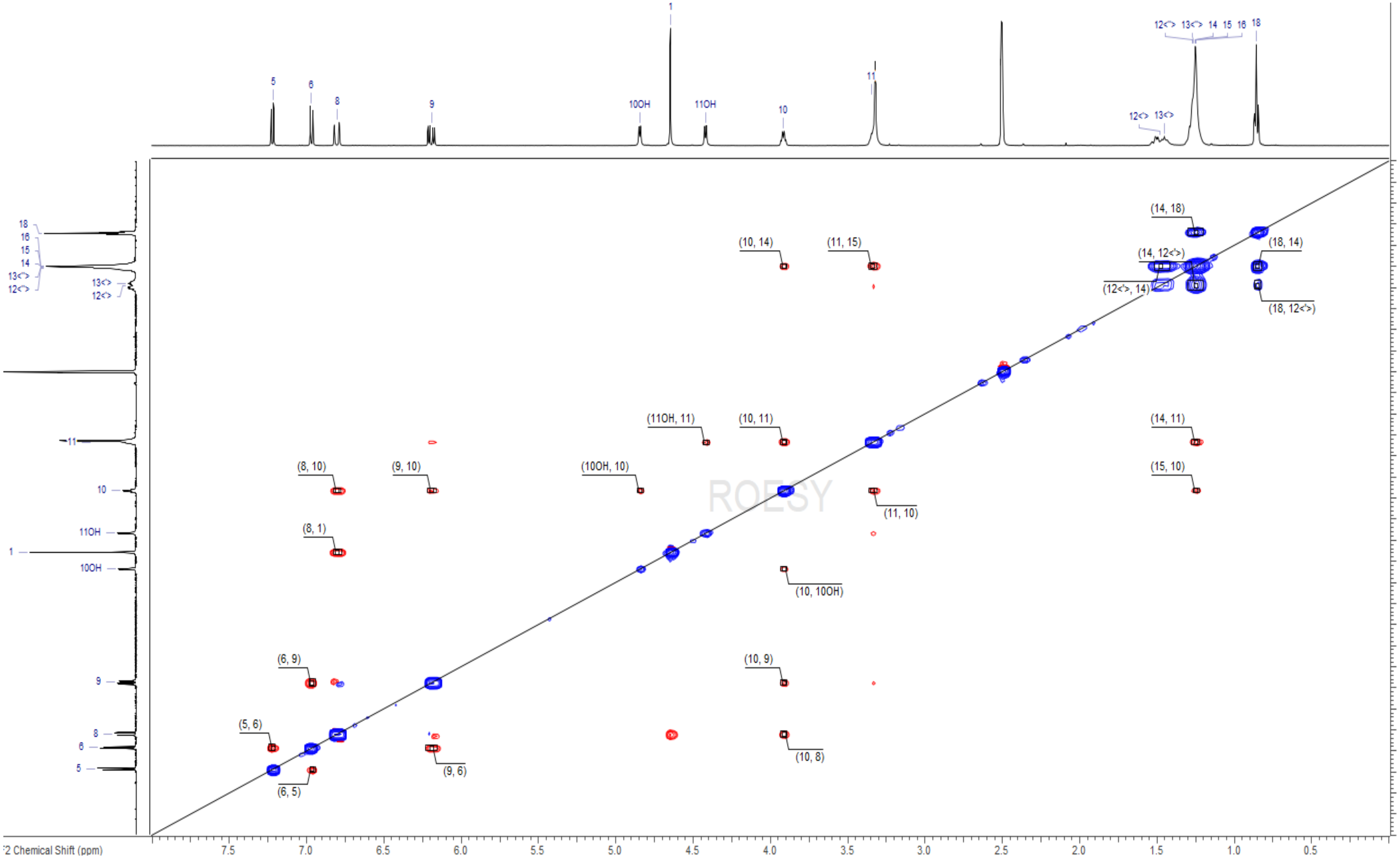

Figure S16. ROESY NMR spectrum (500 MHz, DMSO-d_6) of zopfinol C (**3**).

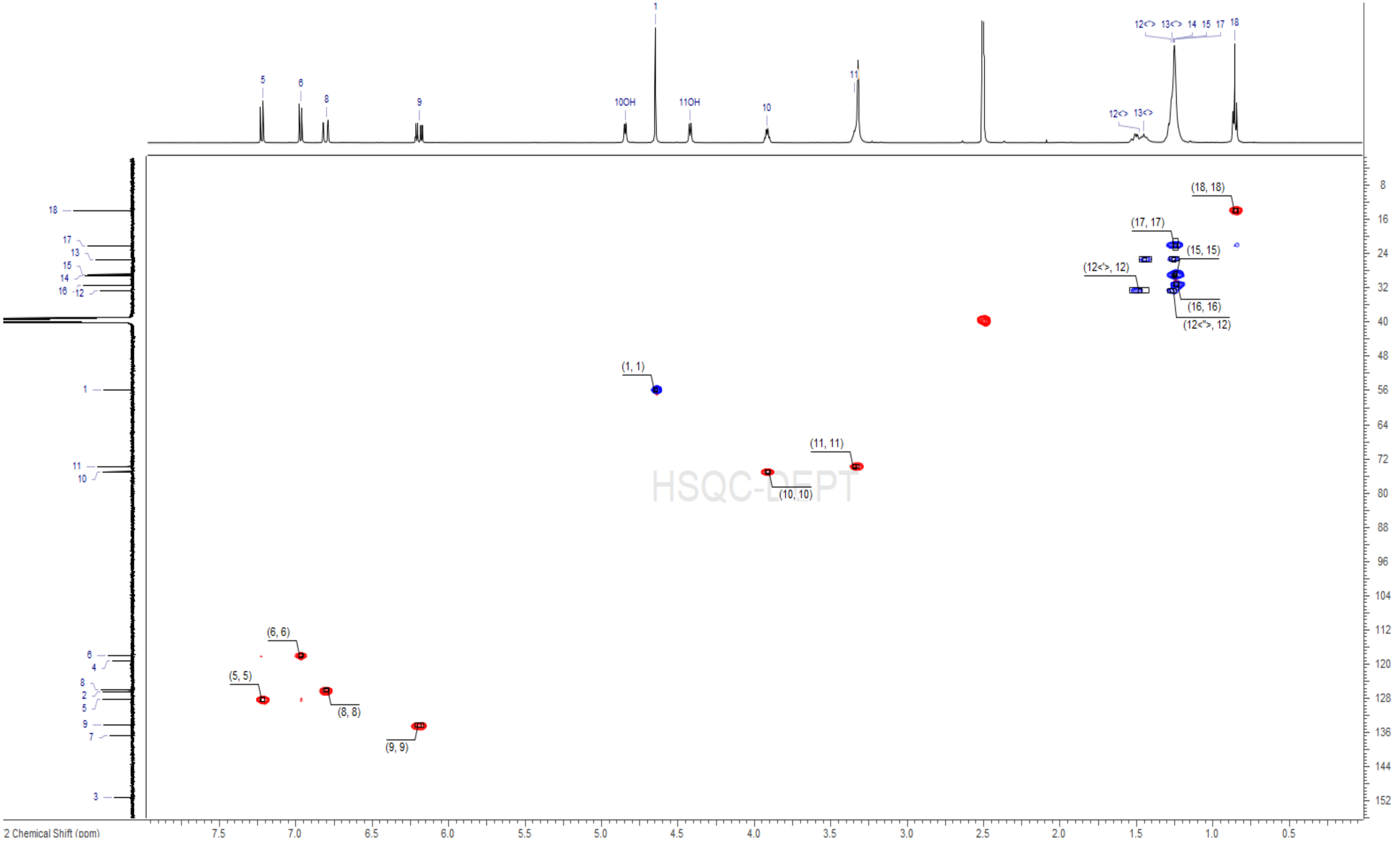

Figure S17. HSQC NMR spectrum (500 MHz, DMSO-d_6) of zopfinol C (**3**).

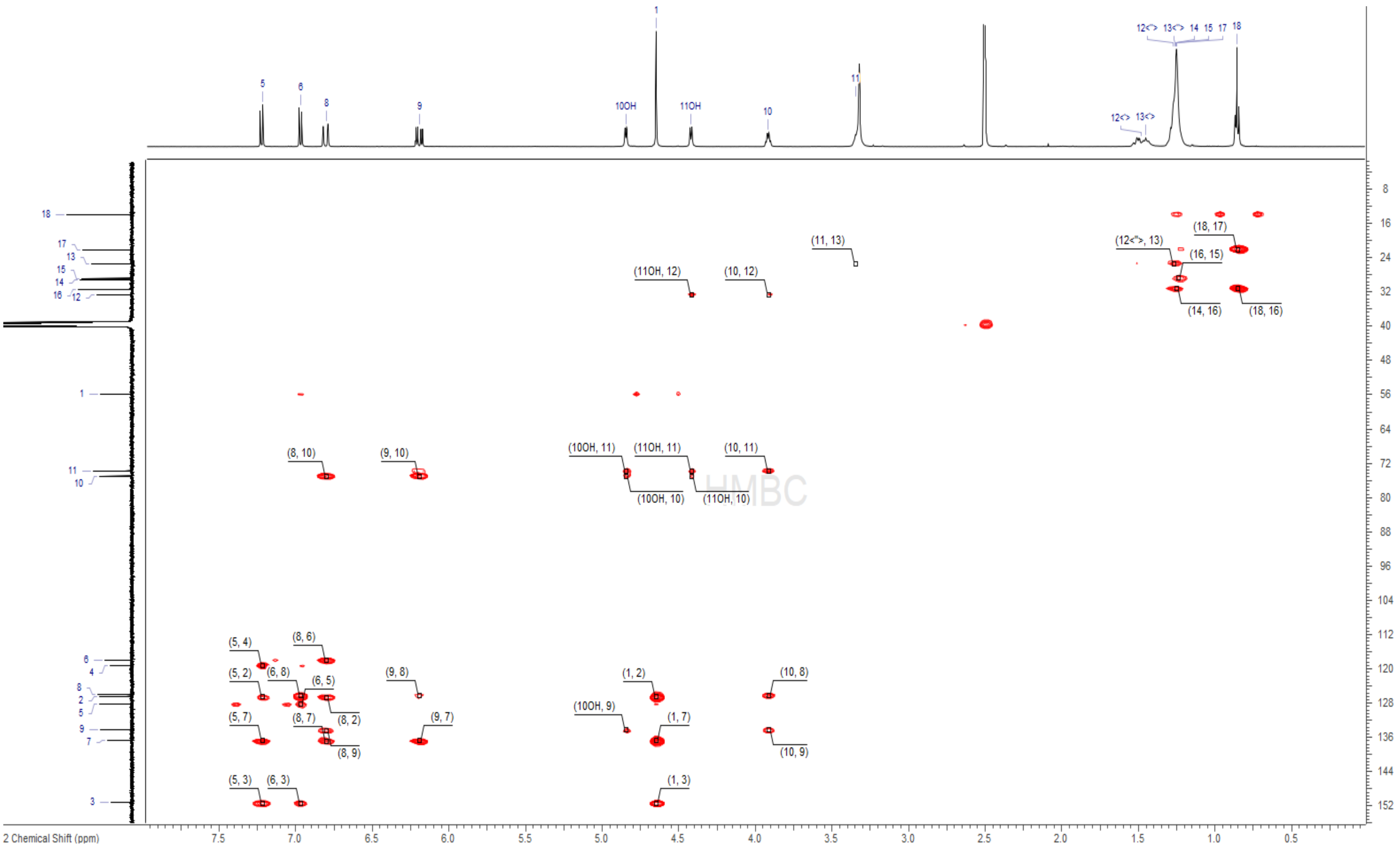

Figure S18. HMBC NMR spectrum (500 MHz, DMSO-d_6) of zopfinol C (**3**).

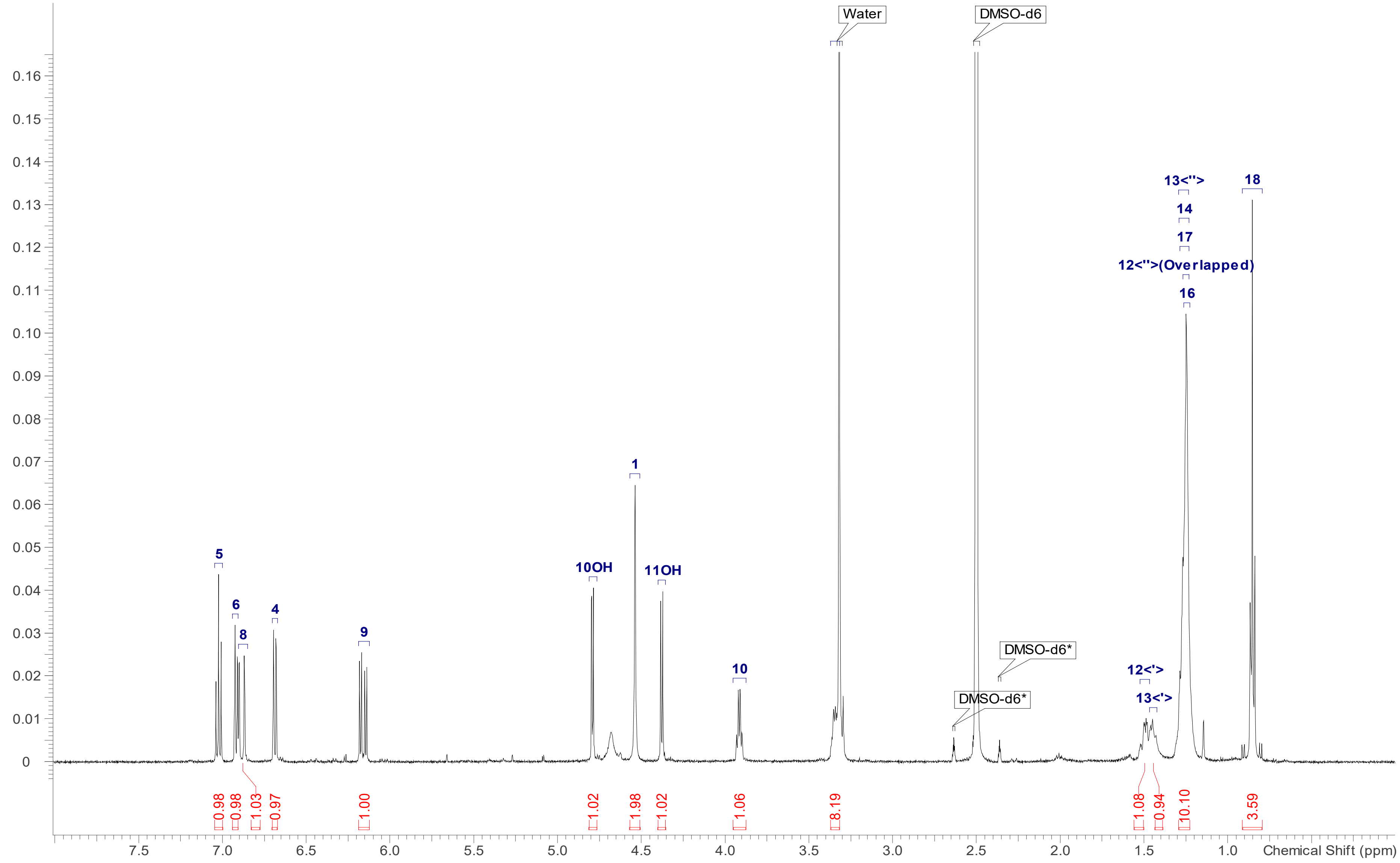

Figure S19. ^{1}H NMR spectrum (500 MHz, DMSO-d_6) of zopfinol D (**4**).

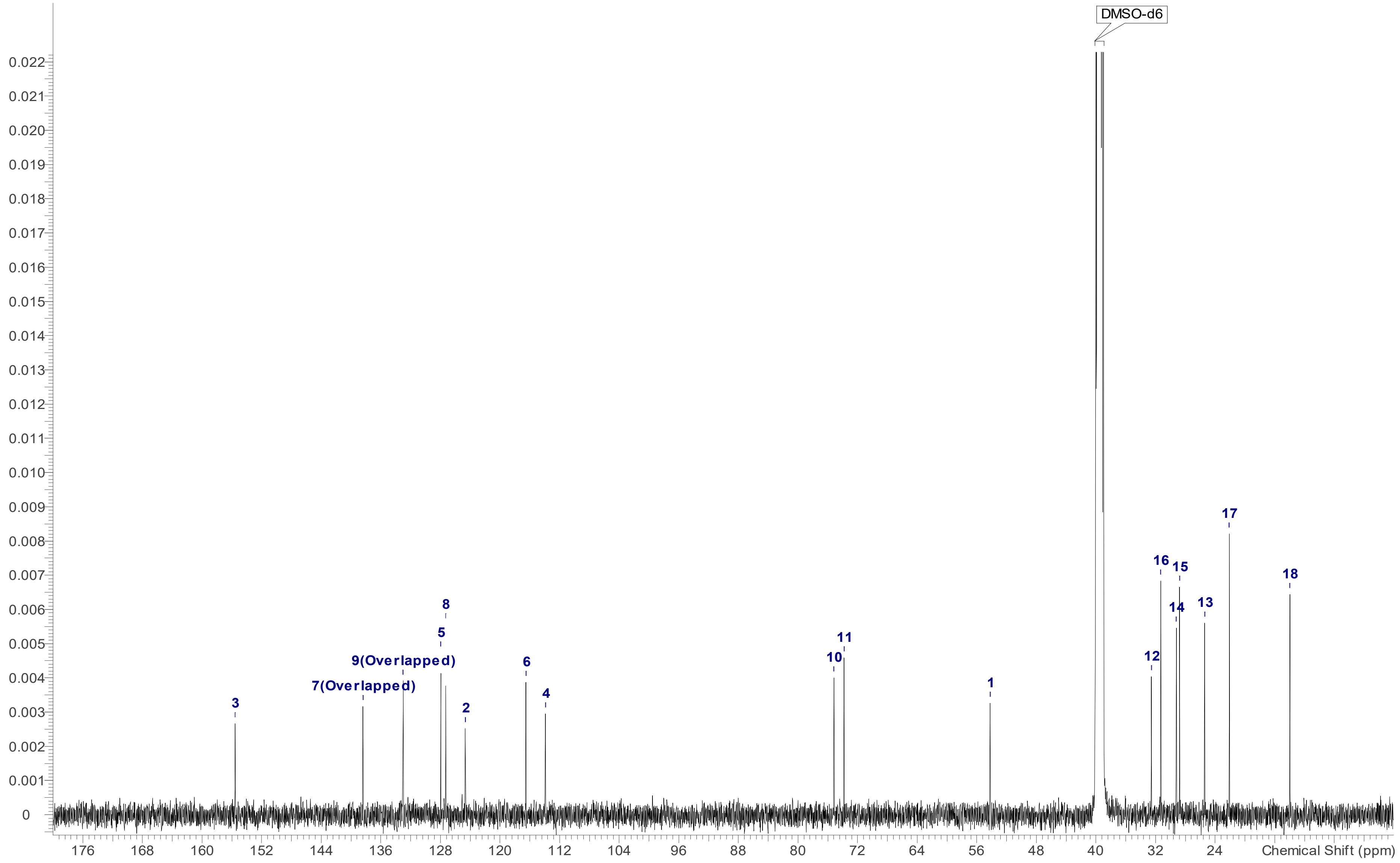

Figure S20. ^{13}C NMR spectrum (125 MHz, DMSO-*d*₆) of zopfinol D (**4**).

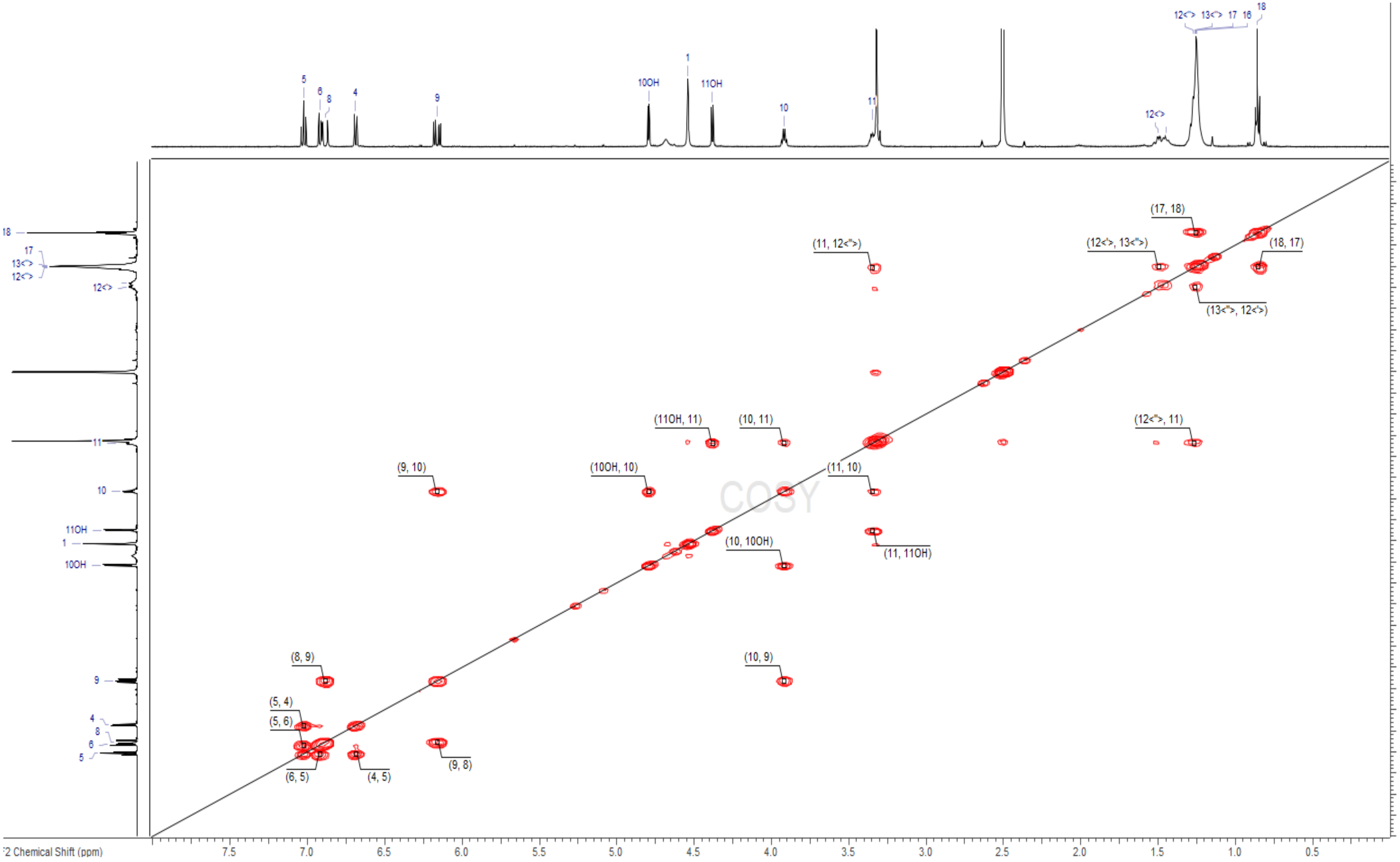

Figure S21. COSY NMR spectrum (500 MHz, DMSO-d_6) of zopfinol D (**4**).

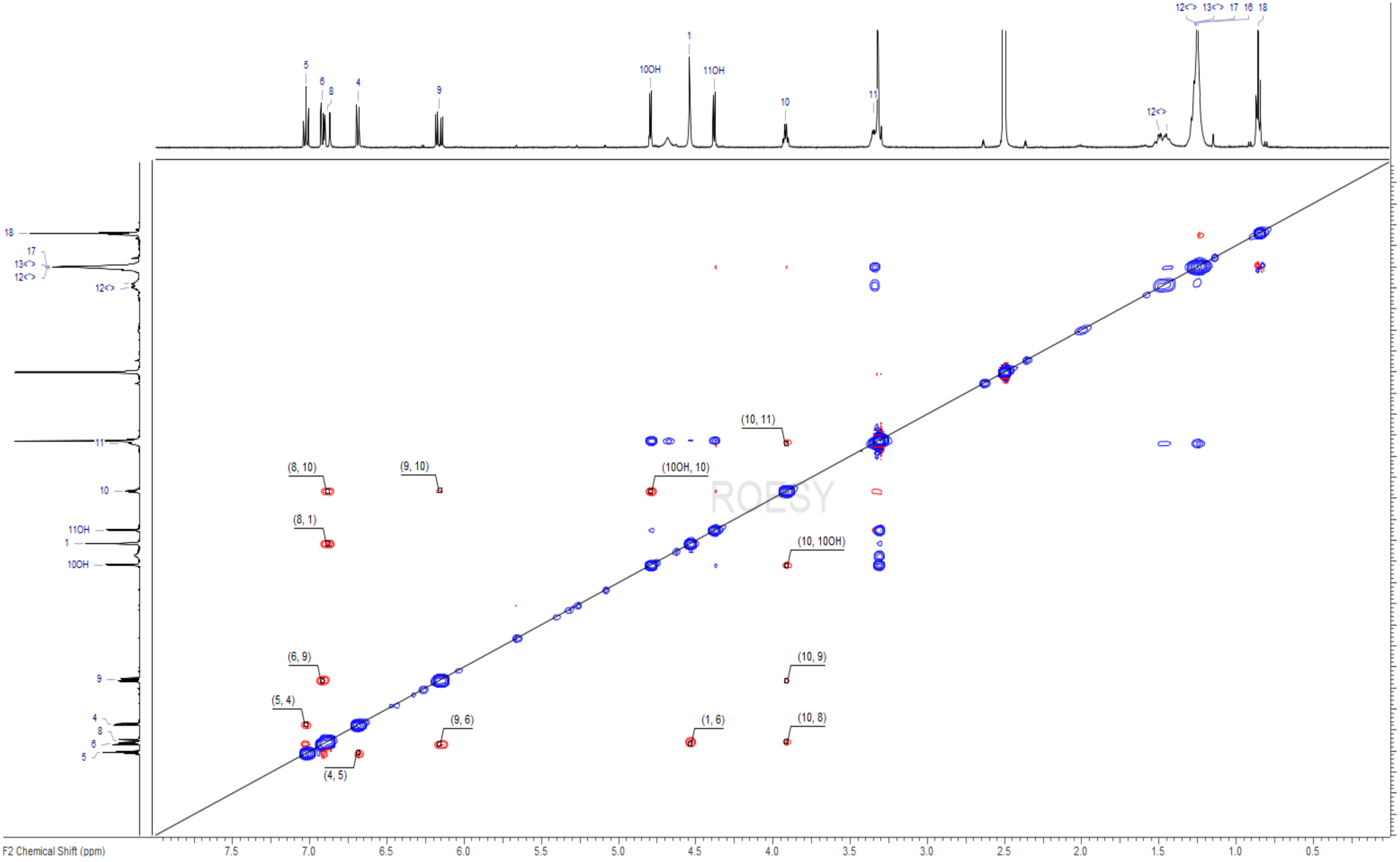

Figure S22. ROESY NMR spectrum (500 MHz, DMSO-d_6) of zopfinol D (**4**).

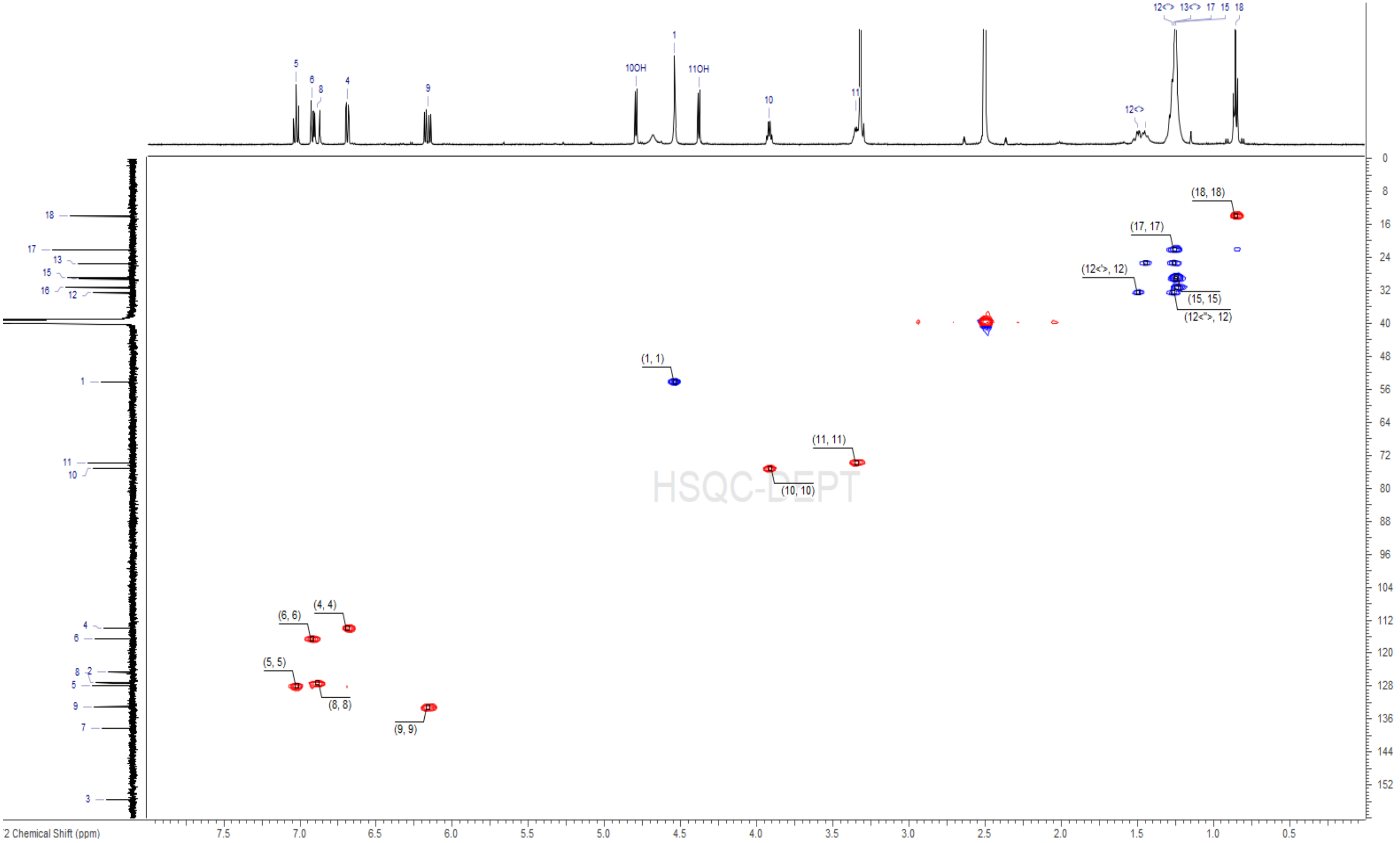

Figure S23. HSQC NMR spectrum (500 MHz, DMSO-d_6) of zopfinol D (**4**).

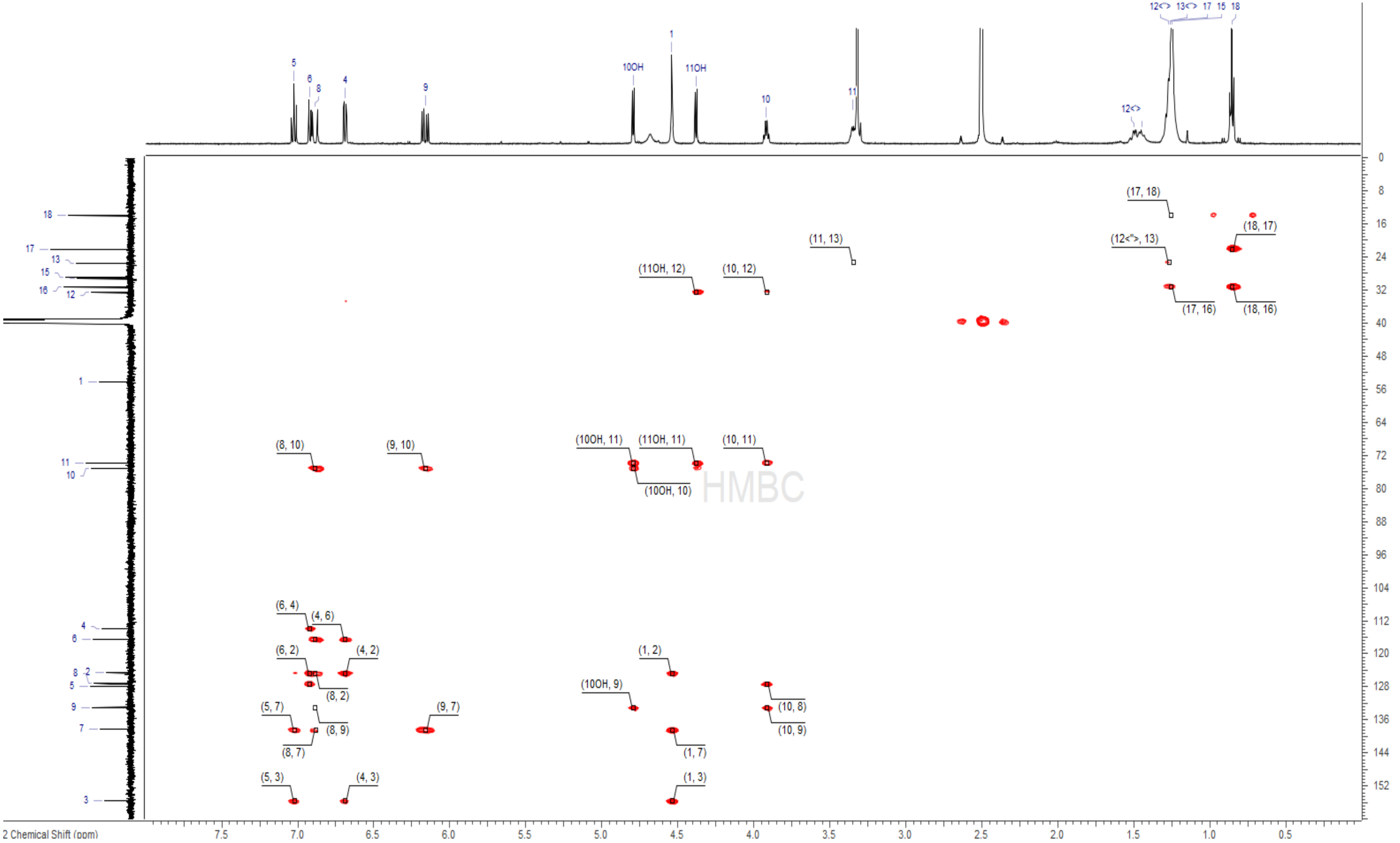

Figure S24. HMBC NMR spectrum (500 MHz, DMSO-d_6) of zopfinol D (**4**).

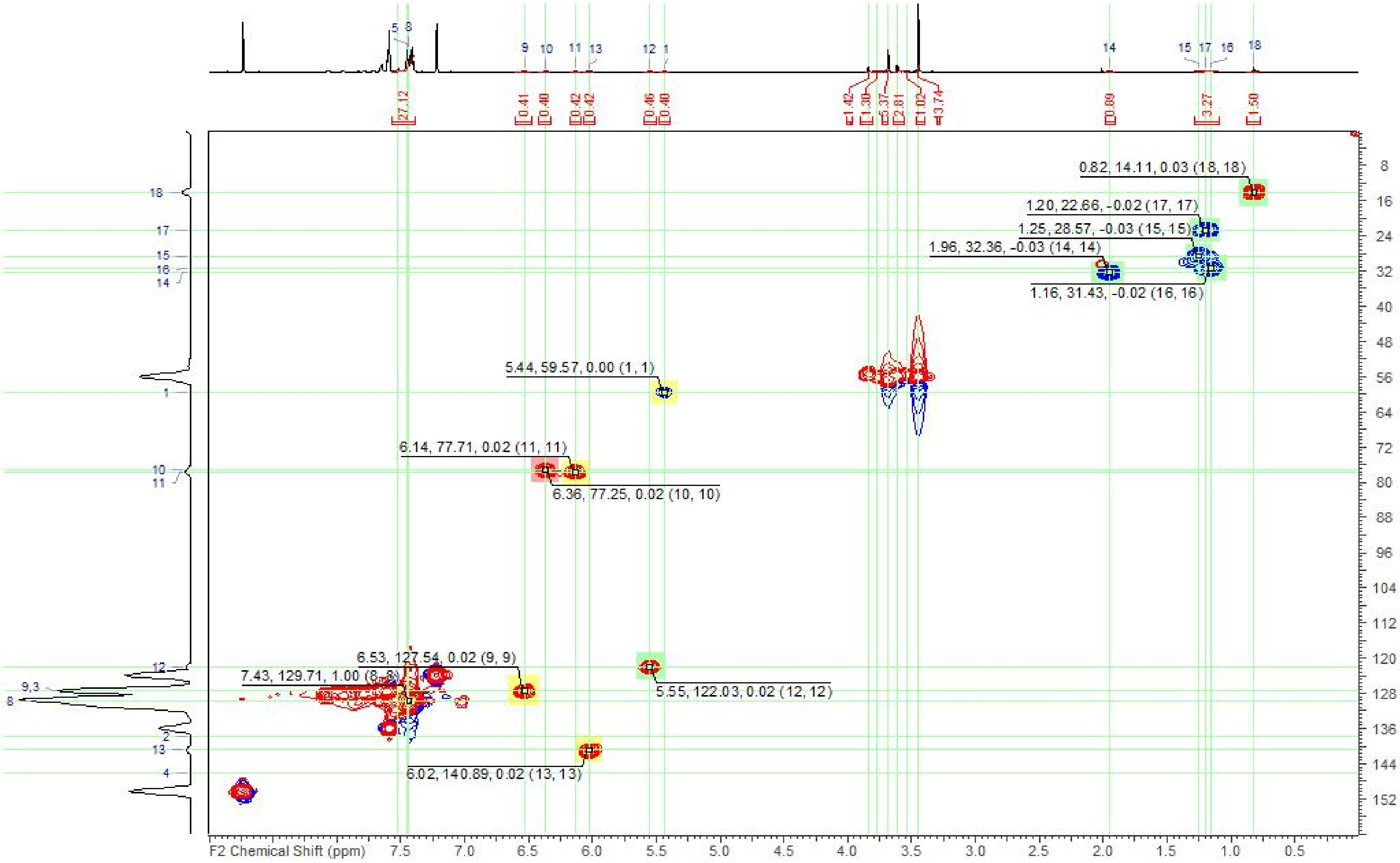

Figure S25. HSQC NMR spectrum (700 MHz, pyridin-d_5) of zopfinol A-*S*-MTPA ester

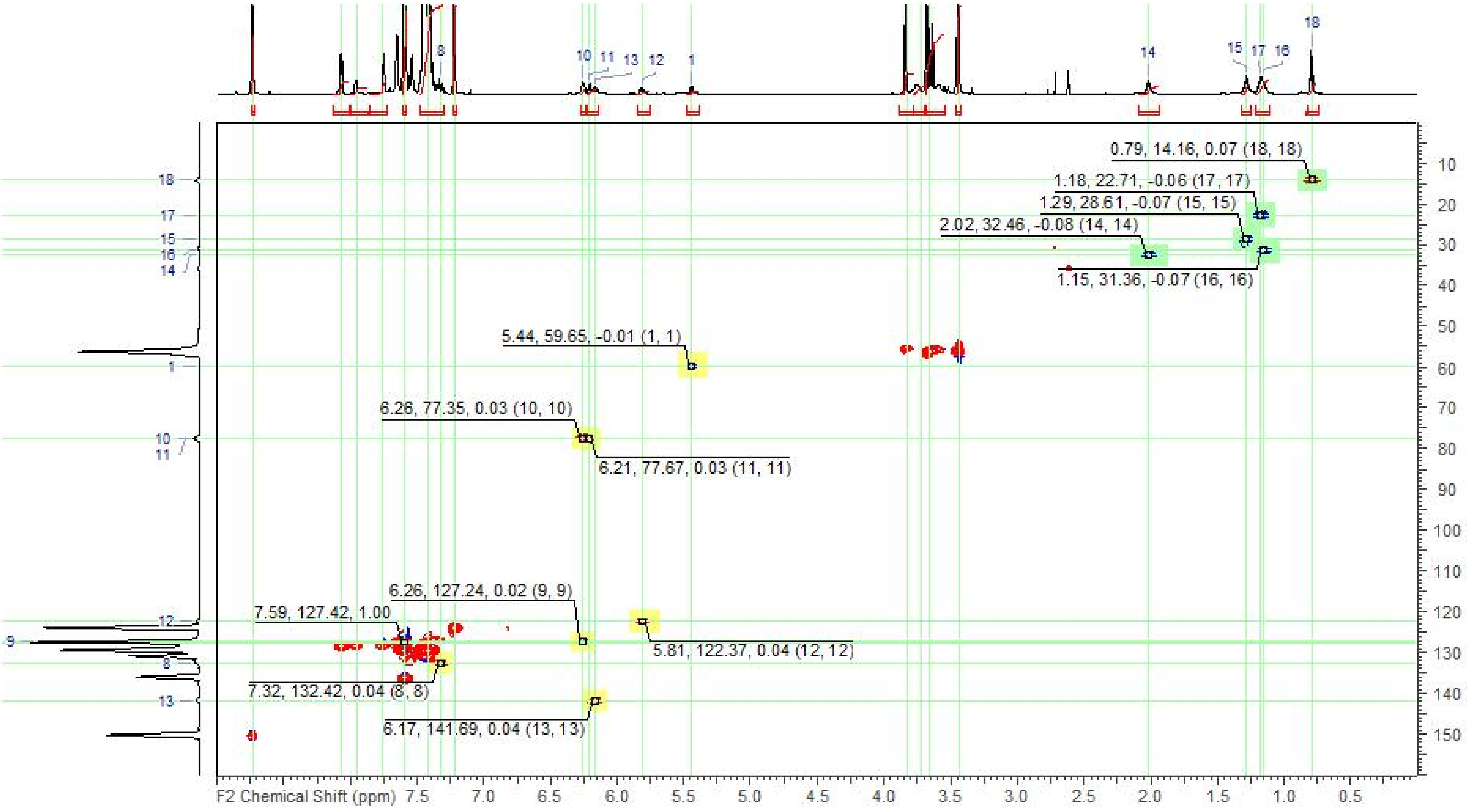

Figure S26. HSQC NMR spectrum (700 MHz, pyridin-d_5) of zopfinol A-*R*-MTPA ester
